# Scorpion Venom Peptides: From Structural Scaffolds to Therapeutic Applications—A Focus on Antioxidant Mechanisms and Translational Perspectives

**DOI:** 10.3390/antiox15060747

**Published:** 2026-06-12

**Authors:** Man Wang, Haoqi Li, Sheng Li, Yanjie Guo, Yijin Xu, Jie Zhao, Lili Chen

**Affiliations:** 1 National-Local Joint Engineering Research Center for Drug-Research and Development (R&D) of Neurodegenerative Diseases, Dalian Medical University, Dalian 116044, China; marina.wang@tjbio.com (M.W.); lihq@dmu.edu.cn (H.L.); lisheng_1996@163.com (S.L.); 13079874269@163.com (Y.X.); 2 School of Basic Medical Sciences, Dalian Medical University, Dalian 116044, China; guoyanjie@dmu.edu.cn

**Keywords:** scorpion venom peptides, oxidative stress, Nrf2-ARE pathway, NADPH oxidase (NOX2), neuroprotection

## Abstract

Scorpion venom peptides, with their stable disulfide backbone, compact structural framework, and highly selective regulation of ion channels, have long been regarded as important molecular probes in neuropharmacology. However, recent studies have revealed their potential for regulating oxidative stress, inflammation, and neuroprotection, making them a new research frontier. In this article, we focus on scorpion venom peptides as drugs, constructing an integrated knowledge framework from structural classification to clinical translation. First, scorpion venom peptides are systematically classified based on cysteine arrangement patterns and three-dimensional folding topology, and their structure–activity relationships are summarized. Based on this, the molecular mechanisms by which scorpion venom peptides regulate ion channels are systematically analyzed. We review the emerging pharmacological activities of scorpion venom peptides. Of particular note, the representative molecule SVHRSP has shown multi-target synergistic antioxidant and neuroprotective activity in models of Parkinson’s disease. We also systematically evaluate the application of engineering strategies, including cyclisation modification, nanodelivery, recombinant expression, and AI-assisted optimization, to overcome the translational bottlenecks in the development of scorpion venom peptides. However, it should be noted that most SVHRSP-related findings have been reported by a single research group; independent replication, pharmacokinetic characterization, and human efficacy data are still lacking. Its IND approval permits clinical investigation but does not yet constitute proven therapeutic benefit in patients. By integrating molecular structure, redox regulation mechanisms, and translational medicine perspectives, this review aims at providing a theoretical basis and practical pathways for scorpion venom peptides as precision therapeutic molecules for oxidative stress-related diseases.

## 1. Introduction

Animal venom is an important source of bioactive molecules for structural optimization in modern drug development, and some venom-derived compounds have inspired the development of clinically applicable drugs [1]. Among these natural resources, scorpion venom has attracted a great deal of attention due to its diverse range of peptide compounds with high targeting specificity, compact conformation, and potent pharmacological activity. In particular, scorpion venom peptides possess a stable disulfide backbone, well-defined ion channel affinity, and significant functional diversity, making them ideal templates for peptide-based drug development. Scorpion venom peptides are secreted by specialized venom glands and are typically small molecules with molecular weights between 1 and 10 kDa. Many scorpion venom peptides are rich in cysteine, and their three-dimensional conformation is stabilized by multiple disulfide bonds; others are linear, non-disulfide peptides with amphiphilic or membrane-active properties. These structural features endow scorpion venom peptides with a variety of biological functions, including regulating ion channels, recognizing membrane-associated proteins, disrupting microbial membranes, and modulating inflammatory signaling pathways [2,3].

Historically, research on scorpion venom peptides has primarily focused on neurotoxicology and ion channel pharmacology. Many classic scorpion venom peptides have become important molecular probes for elucidating the structure and gating mechanisms of voltage-gated sodium (Na_V_), potassium (K_V_), and calcium (Ca_V_) ion channels. In recent years, advances in venomomics, transcriptomics, and structural biology have greatly expanded this field, revealing that scorpion venom peptides are not limited to classic neurotoxins but also have broader therapeutic potential for pain management, cancer diagnosis and treatment, anti-infective therapy, immunomodulation, and intervention in oxidative stress-related diseases [4,5].

Of particular interest is the emerging role of scorpion venom peptides in oxidative stress-related diseases. Oxidative stress is a crucial pathological basis for Parkinson’s disease, Alzheimer’s disease, epilepsy, cerebral ischemia–reperfusion injury, and tumor progression, characterized by excessive reactive oxygen species (ROS), mitochondrial dysfunction, and persistent activation of inflammatory signals. Previous studies have shown that some scorpion venom peptides can exert cytoprotective effects by inhibiting NADPH oxidase (NOX2) activation, reducing ROS accumulation, blocking the NF-κB/NLRP3 inflammatory axis, and improving mitochondrial homeostasis. This suggests that scorpion venom peptides are not only ion channel ligands but may also be multifunctional therapeutic molecules with both membrane-targeting regulatory and antioxidant activities [6,7].

Despite these advances, the clinical translation of scorpion venom peptides remains challenging. Key limitations include insufficient subtype selectivity, limited metabolic stability, poor tissue delivery, potential immunogenicity, and high manufacturing costs [8]. Therefore, a systematic understanding of their structural principles, molecular mechanisms, and engineering solutions is crucial for the development of therapeutics [9].

While some reviews have summarized certain toxin families or therapeutic applications, significant knowledge gaps remain. First, most previous articles primarily categorized toxins by molecular target (e.g., Na_V_ or Kv toxins), with limited cross-family comparisons based on conserved cysteine skeletons, thus obscuring shared and differentiated recognition strategies among different peptides [10]. Conversion barriers such as poor in vivo stability, immunogenicity risks, and limited blood–brain barrier penetration of scorpion venom peptides have not been fully assessed [9,11]. Recent advances in AI-assisted structure prediction and synthetic biology-supported production have not been fully integrated into the current review framework.

Based on this, this review systematically summarizes the research progress of scorpion venom peptides, focusing on the “structural scaffold, functional mechanism, translational application” framework. This article will focus on discussing the structural diversity of major peptide families and their molecular interactions with targets such as Na_V_, K_V_, and TRP. It will further elucidate their emerging pharmacological activities in areas such as antioxidation, anti-inflammation, antitumor, anti-infection, and neuroprotection, and evaluate engineering strategies such as cyclization modification, nanodelivery, recombinant expression, and artificial intelligence optimization. By integrating perspectives from molecular pharmacology, oxidative stress biology, and translational medicine, this article aims to provide a theoretical foundation and practical pathway for the precise development of scorpion venom-derived peptide drugs.

### 1.1. Review Methodology

This review was a structured narrative review based on a systematic literature search. PRISMA principles informed the search strategy, but this review does not include a PRISMA flow diagram, dual independent screening, risk of bias assessment, or quantitative synthesis, as these elements are required only for formal systematic reviews and meta-analyses. The following electronic databases were searched: PubMed/MEDLINE, Web of Science, Scopus, and Google Scholar. The search period covered January 2015 to April 2026. Search terms included combinations of “scorpion venom peptide”, “scorpion toxin”, “CSα/β”, “CSα/α”, “KTx”, “NaTx”, “antioxidant”, “Nrf2”, “NOX2”, “oxidative stress”, “neuroprotection”, “ion channel”, “Na_V_”, “K_V_”, “TRP”, “peptide engineering”, “drug delivery”, and “clinical translation”. Only peer-reviewed original research articles, reviews, and short communications written in English were included. Preprints and conference abstracts were excluded. For mechanistic claims, priority was given to studies with orthogonal validation (e.g., pharmacological inhibition plus genetic knockdown). For translational claims, priority was given to studies reporting quantitative pharmacokinetic or toxicological data.

### 1.2. Scorpion Venom: Biological Context and Peptide Diversity

Scorpions are among the oldest terrestrial arthropods, belonging to the class Arachnida and order Scorpiones. With over 2500 described species distributed across all continents except Antarctica, scorpions have successfully adapted to diverse ecological niches ranging from tropical rainforests to arid deserts. Their evolutionary success is partly attributed to the potent venom they produce, which serves a dual purpose: prey capture and defense against predators [12].

#### 1.2.1. Venom Gland and Production

Scorpions possess a specialized venom apparatus located in the terminal segment of the metasoma, commonly known as the telson. The telson contains a pair of symmetrical, sac-like venom glands connected to a stinger (aculeus) through which venom is injected. Venom biosynthesis occurs in specialized secretory cells lining the gland epithelium, where peptide precursors are synthesized, processed through post-translational modifications (including disulfide bond formation, amidation, and pyroglutamylation) and stored in the gland lumen prior to use [13].

#### 1.2.2. Venom Composition

Scorpion venom is a complex biological cocktail containing a wide array of bioactive molecules. The primary components are peptides and small proteins (typically 1–10 kDa), which constitute the majority of the venom’s dry weight and are responsible for its pharmacological effects. To peptides, scorpion venom also contains enzymes (e.g., hyaluronidase, phospholipase, and protease), mucoproteins, biogenic amines (e.g., serotonin, and histamine), salts, and other small organic molecules [14,15].

#### 1.2.3. Classification of Venom Peptides

Scorpion venom peptides are most commonly classified based on their molecular targets and biological functions. Neurotoxins: These are the most abundant and well-studied components. They target voltage-gated ion channels, including sodium (Na_V_), potassium (K_V_), calcium (Ca_V_), and chloride (ClC) channels. Based on their channel specificity and mode of action, neurotoxins are further divided into α- and β-toxins (Na_V_ channel modulators), α-KTx peptides (K_V_ channel blockers), and other less abundant families [16]. It should be noted that “α-KTx” is a functional/sequence-based classification (based on potassium channel blocking activity and sequence homology), not a structural one. Members of this classification adopt different three-dimensional scaffolds: most α-KTx subfamilies (e.g., α-KTx1.x, α-KTx2.x, α-KTx3.x) adopt a CSα/β scaffold, while the κ-KTx subfamily (α-KTx6.x) adopts a CSα/α scaffold. This distinction is discussed in detail in Section 2.1.2. Cytotoxins: These peptides exert direct cytotoxic effects on various cell types. Cardiotoxins and hemolytic toxins belong to this category, although they are less common in scorpion venoms compared to those of snakes or spiders. Antimicrobial peptides (AMPs): Many scorpion venoms contain linear or weakly disulfide-bonded peptides with broad-spectrum activity against bacteria, fungi, and even viruses. These peptides typically adopt amphipathic α-helical conformations upon membrane contact and are considered promising templates for antibiotic development [15]. Enzymatic and other components: Hyaluronidase (a spreading factor), phospholipase, and other hydrolytic enzymes facilitate venom diffusion and tissue penetration during envenomation [17]. This molecular diversity has made scorpion venom an invaluable natural resource for discovering new pharmacological tools and therapeutic leads, as discussed in the following sections.

## 2. Structural Classification and Molecular Architecture

**Figure 1:**
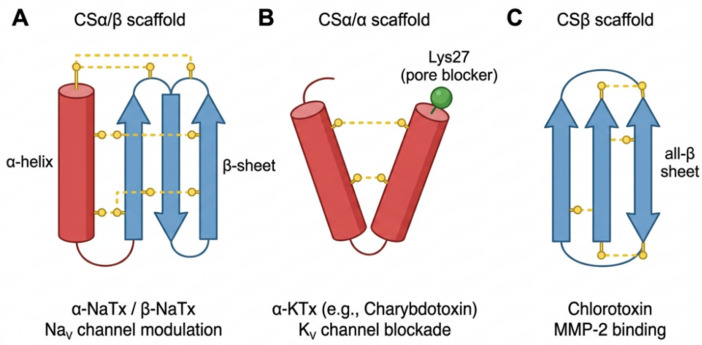
Topological schematic of the three major structural scaffolds of scorpion venom peptides. This figure illustrates the three major structural scaffolds of scorpion venom peptides. The CSα/β scaffold (**A**) consists of an α-helix tightly packed against a three-stranded antiparallel β-sheet, typically stabilized by four disulfide bonds; it is predominantly found in sodium channel-targeting toxins (α-NaTx and β-NaTx). The CSα/α scaffold (**B**) comprises two short α-helices stabilized by disulfide bonds, forming a “two-finger” topology characteristic of the κ-KTx subfamily of potassium channel inhibitors. The CSβ scaffold (**C**), represented by chlorotoxin, is composed of three antiparallel β-sheets with no α-helical content, enabling high-affinity binding to MMP-2 and annexin A2 for glioma targeting. These structurally distinct scaffolds provide the molecular basis for the functional diversity of scorpion venom peptides. Refer to Section 2.1.1, Section 2.1.2 and Section 2.1.3. Abbreviations: CSα/β, cysteine-stabilized α-helix/β-sheet; CSα/α, cysteine-stabilized α-helix/α-helix; CSβ, cysteine-stabilized β-sheet; MMP-2, matrix metalloproteinase-2.

The structural diversity of scorpion venom peptides underlies their functional specificity. Based on the arrangement of cysteine residues and disulfide bond linkages, scorpion venom peptides can be systematically classified into several structural families, among which the CSα/β (cysteine-stabilized α-helix/β-sheet), CSα/α, and CSβ families constitute the core architecture of the venom group [18] (Figure 1). These compact structural motifs endow venoms with high thermal stability, proteolytic resistance, and evolutionary adaptability, making them common templates for venom diversification. To these disulfide-rich peptides, scorpion venom also contains linear non-disulfide peptides with membrane activity or antibacterial properties. These peptide families together constitute a structurally coherent yet functionally diverse group of venom peptides. This section summarizes the main classification systems of scorpion venom peptides, their characteristic three-dimensional folded structures, and the latest advances in structural biology, which have deepened our understanding of the skeleton-function relationship (Table 1).

### 2.1. Cysteine-Based Scaffold Classification System

#### 2.1.1. CSα/β Family: Classic α-Helix β-Fold Double-Chain Structure

The CSα/β sheet is the most widely distributed structural motif in scorpion venom peptides. It is typically stabilized by four disulfide bonds formed by eight cysteine residues and consists of a tightly packed α-helix and a triple-stranded antiparallel β-sheet [18]. This scaffold structure is dominant in sodium channel toxins and contains two main functional groups: α-scorpion venom (α-NaTxs) and β-scorpion venom (β-NaTxs).

α-NaTxs (such as AaH II) bind to receptor site 3 located on the extracellular S3–S4 loop of domain IV (DIV) of voltage-gated sodium (Na_V_) channels. This binding restricts the outward movement of the DIV voltage sensor S4, delaying fast inactivation and prolonging sodium current duration. A representative member is AaH II from the North African scorpion (*Androctonus australis*; also known as the Sahara scorpion). Conversely, β-NaTxs (such as CsVII from the Arizona bark scorpion, *Centruroides sculpturatus*) bind to receptor site 4 located on the S3–S4 paddle motif of domain II (DII) of Na_V_ channels. This binding stabilizes the voltage sensor in an activated conformation, shifting the channel activation curve toward hyperpolarized potentials and lowering the threshold for channel opening. Although the pharmacological effects of α- and β-toxins differ, crystallographic and NMR studies indicate that they share a conserved CSα/β core structure, with functional differences primarily depending on surface electrostatic interactions and conformational changes in the exposed ring, which participate in receptor recognition. Recent studies have further highlighted the adaptability of this scaffold. The newly discovered β-toxins Chirp7 and Chirp9 from Centruroides hirsutipalpus (hirsute-palped scorpion) interact with the S3–S4 paddle motif of Na_V_ channels, providing new structural templates for the development of channel-selective probes and modulators [19].

#### 2.1.2. CSα/α Family: Double-Helix Scaffold for κ-KTx Peptides

Scorpion venom peptide toxins targeting voltage-gated potassium channels (K_V_) can be structurally divided into two distinct families: the CSα/β family (most classic α-KTx peptides) and the CSα/α family (κ-KTx peptide subfamily).

Most classic scorpion potassium channel toxins (including charybdotoxin, Iberian toxin, magatoxin, and agitoxin-2) actually employ a CSα/β scaffold, a compact structure formed by the close stacking of an α-helix and an antiparallel β-sheet via disulfide bonds [20]. Although these toxins are similar in overall folding to sodium channel-targeting CSα/β toxins, their functional surfaces and loop structures have evolved to specifically recognize the outer pore regions of K_V_ channels. For example, charybdotoxin (α-KTx1.1) directly and physically blocks potassium ion permeation by inserting its functional lysine residue (Lys27) into the channel pores [21]. These venoms should be classified structurally as belonging to the CSα/β family, not the CSα/α family.

In contrast, the κ-KTx subfamily (such as HsTx1 and BmTx3) employs a typical CSα/α scaffold, consisting of two short, stable α-helices linked by disulfide bonds, forming a “two-finger” or “three-finger” topology [18]. κ-KTx peptides achieve allosteric inhibition rather than direct physical pore blockage by binding to the extracellular surface of K_V_ channels and adjacent gating domains, stabilizing the non-conductive state of the channels. HsTx1 (κ-KTx1.1 or α-KTx6.2) is a representative member of this family, exhibiting picomolar inhibitory activity against K_V_1.3, and has become an important template for optimizing immunomodulatory peptides [22].

Currently, more than 30 sequence-based α-KTx subfamilies have been identified. However, it should be noted that “α-KTx” is a classification system based on function (potassium channel blocking activity) and sequence homology, rather than on three-dimensional folding topology. Different members within the same α-KTx subfamily may employ different structural scaffolds. For example, the α-KTx1.x subfamily (charybdotoxin and Iberiotoxin) uses a CSα/β scaffold, while the α-KTx6.x subfamily (HsTx1 and BmTx3) belongs to the CSα/α scaffold [18]. Therefore, when discussing structure function relationships, it is important to clearly distinguish their three-dimensional folding types.

To avoid confusion between functional nomenclature and structural classification, it is important to recognize that “α-KTx” is a functional/sequence-based classification system based on potassium channel blocking activity and sequence homology. This system includes members from two distinct structural families: most α-KTx subfamilies (e.g., α-KTx1.x, α-KTx2.x, α-KTx3.x) adopt a CSα/β scaffold, while the κ-KTx subfamily (also referred to as α-KTx6.x, including HsTx1 and BmTx3) adopts a CSα/α scaffold. Therefore, when discussing structure-function relationships, one should refer to the three-dimensional scaffold type rather than relying solely on the α-KTx nomenclature.

#### 2.1.3. CSβ Family: Chlorotoxin-like Disulfide-Rich Peptides

The CSβ family is represented by chlortoxin, a compact peptide stabilized by a C-C-C-C framework of four cysteine residues. Its overall folding consists primarily of three antiparallel β-sheets, lacking the typical α-helix. This structure enables it to form high-affinity interactions with membrane-associated targets, including matrix metalloproteinase-2 (MMP-2) and annexin A2, which explains its long-standing interest as a glioma-targeting ligand in imaging and drug delivery applications [23]. The structural diversity of chlortoxin-like peptides continues to expand. In 2025, researchers identified a novel chlortoxin-like insecticidal peptide, Ce1, from the venom of the Egyptian scorpion (*Compsobuthus egyptiensis*). Ce1 consists of 36 amino acid residues and four disulfide bonds, but unlike typical chlortoxins, it employs a mixed folding structure consisting of one α-helix and three antiparallel β-sheets. This discovery broadens the known structural spectrum of the CSβ family and provides a new evolutionary solution for selective predatory toxicity [24].

#### 2.1.4. Cysteine-Free Linear Peptides: Antimicrobial and Membrane-Active Peptides

To disulfide-rich toxins, scorpion venom also contains a large number of cysteine-free linear peptides. A typical example is Pandinin 1 from the emperor scorpion (Pandinus imperator), which exerts its antibacterial activity by disrupting microbial cell membranes through the formation of amphiphilic α-helices. A recent study comparing Pandinin 1, Pandinin 2, and Pandinin 3 against multidrug-resistant Klebsiella pneumoniae showed that Pandinin 1 and Pandinin 2 produced rapid bactericidal effects within one hour, with minimum inhibitory concentrations of 6–25 μM. Mechanistic analysis indicated that Pandinin 1, Pandinin 2, and Pandinin 3 directly interact with lipopolysaccharides in the bacterial outer membrane [25]. Short-chain linear scorpion venom peptides typically contain 9–19 amino acid residues and are attracting increasing attention due to their ease of synthesis and relatively low production costs. These molecules, along with disulfide-rich toxins, demonstrate two complementary evolutionary strategies in scorpion venom: broad-spectrum membrane defense and highly selective receptors and highly selective targeting of receptors and ion channels.

#### 2.1.5. Comprehensive Venom Peptide Components

The peptide families summarized in Table 1 represent the major structural classes of scorpion venom peptides that have been functionally and structurally characterized. However, it is important to note that high-throughput venomomics and transcriptomic analyses have revealed hundreds of additional venom components from diverse scorpion species, many of which remain functionally uncharacterized. Readers seeking comprehensive listings of all known scorpion venom peptides are referred to specialized databases: UniProt (https://www.uniprot.org), and the VenomZone portal (https://venomzone.expasy.org, accessed on 15 March 2026).

### 2.2. Three-Dimensional Structural Features and Dynamic Properties

#### 2.2.1. Conformational Constraints of Disulfide Bonds

Disulfide bonds are key determinants of the folding and stability of scorpion venom peptides. In the classic cysteine-stabilized α/β (CSα/β) motif, two disulfide bonds link the α-helix to the C-terminal antiparallel β-sheet, forming the most common compact scaffold in scorpion venom. A complete CSα/β fold typically contains three disulfide bonds, at least two of which anchor the helix to the β-sheet, forming a rigid hydrophobic core. Comparative studies have shown that this scaffold is not unique to scorpions and is also conserved in insect defensins and plant γ-sulfur proteins, highlighting its evolutionary success as a functional template [26]. However, disulfide bond connections are not absolutely conserved. Saucedo et al. described two potassium channel-blocking peptides from the scorpion Tityus that retain the typical six-cysteine spacing but employ different pairing patterns. This rearrangement is accompanied by a complete recombination of secondary structures, forming a cysteine-stabilized helix-loop-helix fold, revealing unexpected structural plasticity in the venom repertoire [27]. Atypical pairing patterns have also been reported in some centruroid sodium channel toxins, further expanding the known structural map.

A complete disulfide bond network also significantly contributes to thermostability. The degradation-derived peptide BmK86-P1 isolated from medicinal scorpion material retains its native fold over a wide temperature range (20–95 °C), indicating strong thermostability, which is closely related to the preservation of disulfide bond connections [28]. Mutational analysis of leucine toxin I further shows that the loss of key disulfide bonds reduces α-helix content and bioactivity. Although two native bridges still maintain a near-native folded structure, all three disulfide bonds are essential for complete thermodynamic stability [26].

#### 2.2.2. Functional Surfaces and Molecular Recognition

Scorpion venom recognition of ion channels depends on finely arranged surface properties. For sodium channel toxins, α-toxins and β-toxins bind to different receptor sites: α-toxins interact with receptor site 3 and inhibit rapid inactivation, while most β-toxins bind to receptor site 4 and shift voltage-dependent activation to a more hyperpolarised potential [29]. Electrostatic analysis shows that many sodium channel toxins exhibit asymmetric charge distributions. Positively charged regions (typically with theoretical isoelectric points above 8.5) promote initial attraction to negatively charged residues in voltage-sensing domains or extracellular loops, while hydrophobic regions stabilize subsequent docking. Crystal structure analysis of Ts1 revealed a single-sided cluster of residues, previously confirmed by chemical modification and mutagenesis, defining a possible channel recognition surface where charged and nonpolar side chains jointly determine selectivity [30].

#### 2.2.3. Post-Translational Modifications Expand Chemical Diversity

Scorpion peptides undergo various post-translational modifications (PTMs), significantly broadening their chemical and functional range. C-terminal amidation is one of the most common modifications, crucial for both ion channel toxins and antimicrobial peptides. Transcriptomic analysis of the venom glands identified a dual-enzyme α-amidation system consisting of a membrane-bound bifunctional peptidylglycine α-amidation monooxygenase and an associated soluble enzyme, providing the molecular basis for scorpion peptide amidation [13]. Functionally, C-terminal amidation enhances the activity of various toxins. In the BmKAS of *Mesobuthus martensii*, amidation increases its affinity for insect voltage-gated sodium channels [31]. A similar phenomenon was observed in the Ts1 of *Tityus serrulatus*, where amidation affects its biological activity and proteolytic stability [32]. N-terminal pyroglutamylation is another important post-translational modification. Ts1 (also known as γ-toxin) is a typical example. Although pyroglutamylation is less frequent in scorpions than in cone snails or snake venom, it can protect the N-terminus from exopeptidases and regulate its interaction with targets [33]. A review of engineered scorpion antimicrobial peptides further indicates that C-terminal amidation and specific proline substitutions can enhance protease resistance and stabilize amphiphilic helical structures, while hydroxyproline may enhance local conformational preference [34].

### 2.3. Advances in Structural Biology Methodology

#### 2.3.1. X-Ray Crystallography and Nuclear Magnetic Resonance Spectroscopy

X-ray crystallography remains a primary method for determining high-resolution three-dimensional structures of scorpion venom peptides. The Protein Data Bank (PDB) was queried on 15 March 2026 using the keywords “scorpion toxin” or “scorpion venom peptide”. The structures discussed below include experimentally determined scorpion venom peptides (isolated toxins and toxin-channel complexes). Predicted models generated by artificial intelligence tools such as AlphaFold2/3 are not included, nor are non-scorpion comparative structures (e.g., spider toxins), which are discussed separately as comparative models in Section 2.3.2.

X-ray crystallography has provided high-resolution three-dimensional structures of many scorpion venom peptides and their complexes with ion channels. Representative examples include toxin II from *Androctonus australis* Hector solved at 0.96 Å (PDB: 1AHO) and BmBKTx1 from *Mesobuthus martensii* determined at 1.10 Å (PDB: 3E8Y). These structures have offered atomic-level insights into disulfide frameworks and folds. Nuclear magnetic resonance (NMR) spectroscopy has also contributed extensively to the field, capturing the dynamic behavior of scorpion toxins in solution. NMR is particularly valuable for studying conformational flexibility, such as the picosecond to nanosecond timescale motions of functional loop regions in BmKK2, which are critical for understanding toxin-membrane interactions [35]. Cryo-electron microscopy (cryo-EM) has become a powerful tool for visualizing scorpion venom peptides in complex with their membrane protein targets, including complexes of α-scorpion toxins with sodium channels, revealing the molecular details of toxin-channel recognition and gating modulation. These cryo-EM structures have revealed how scorpion toxins recognize voltage-sensing domains and modulate channel gating. Predicted models generated by artificial intelligence tools such as AlphaFold2/3 are not experimentally determined structures. While these predictions have advanced the field, they require experimental validation, particularly for toxin-channel interfaces involving glycosylated or highly flexible extracellular loops.

#### 2.3.2. The Cryo-Electron Microscopy Structural Revolution

Since the “resolution revolution” of 2017, cryo-electron microscopy has become a core technology for resolving the structure of scorpion venom peptide membrane protein complexes. In 2019, Clairfeuille et al. [36] resolved the complex structure of α-scorpion venom LqhIII and the Na_V_1.5 channel at a resolution of 3.3 Å, demonstrating for the first time at the atomic level how α-scorpion venom binds to the S3–S4 linker region of the voltage-sensing domain IV, exerting its effect by hindering channel inactivation. By designing a Na_V_Ab/Na_V_1.7 chimeric channel in 2021, Wisedchaisri et al. resolved the structural details of Huwentoxin-IV (a tarantula venom peptide, included here as a comparative structural model, not as a scorpion-derived peptide), capturing the resting state of Na_V_1.7 at a resolution of 2.8 Å and revealing that key lysine residues in the venom act as “stingers”, penetrating deep into the S3–S4 linker region of the voltage receptor and locking the channel in a resting state [37]. This research validates and extends the gated modified toxin action model proposed in electrophysiological studies, providing an atomic-level template for the rational design of ion channel-targeted drugs.

#### 2.3.3. Computational Structural Biology and Artificial Intelligence

Molecular dynamics simulations effectively compensate for the lack of dynamic information in static experimental structures. All-atom molecular dynamics (MD) simulations revealed the stepwise insertion behavior of the scorpion venom peptide ADWX-1 during its binding to the K_V_1.3 channel, predicting the transient interactions between aromatic residues of the toxin and the hydrophobic ring in the channel pore region, dynamic details difficult to capture directly through crystallography [38]. The release of AlphaFold2 in 2021 revolutionized the scorpion venom peptide structure prediction paradigm: its prediction confidence (pLDDT > 90) for the CSα/β and CSα/α family toxin backbones approached the precision of NMR experiments [39]. In 2024, AlphaFold3 further achieved end-to-end prediction of toxin–protein complexes, but its confidence in predicting the extracellular loop region of channels with multiple glycosylation sites remains low, a key region for scorpion venom peptide binding. Therefore, its applicability to ion channel complexes containing multiple transmembrane helices still needs systematic validation with experimental data [40].

### 2.4. An Integrated Perspective on Structure Function Relationships

The CSα/β and CSα/α scaffolds of scorpion venom peptides exhibit profound modularity in both evolution and function. Although they share a similar disulfide bond core, they achieve distinctly different target recognition strategies through differentiated combinations of secondary structures: the CSα/β scaffold utilizes the functional surface recognition channel between the α-helix and β-sheet, while the CSα/α scaffold mainly relies on the spatial configuration of the double α-helix and the connecting loop region to function. The first insecticidal peptide with a CSα/α scaffold, LaIT5, identified in 2024 from the scorpion *Liocheles australasiae*, has a double α-helix and two consecutive histidine residues that are crucial for its insecticidal activity and are non-toxic to mammals, demonstrating the potential of the CSα/α scaffold as a template for the development of novel biopesticides [41]. At the molecular engineering level, customized functional modification has been successfully achieved through domain transplantation between different CSα/β toxins. In 2025, Montero-Dominguez et al. [42] grafted the domain of CeII8 from non-lethal β-scorpion venom onto the backbone of the lethal β-scorpion venom toxin CssII, yielding the chimeric peptide rCssII-Del-D23A-TCD. This variant retains affinity for Na_V_1.7 but loses toxicity toward Na_V_1.6 and exhibits analgesic activity comparable to the μ-opioid receptor agonist DAMGO in inflammatory and neuropathic pain models [42]. In the future, the deep integration of cryo-electron microscopy, artificial intelligence prediction, and high-throughput mutation screening is expected to accelerate the transformation of scorpion venom peptides from natural molecules into precision therapeutic tools targeting specific ion channel diseases.

It is important to note that “α-KTx” is a classification system based on function (potassium channel blocking activity) and sequence homology, rather than on three-dimensional folding topology. Different members within the same α-KTx subfamily may employ different structural scaffolds. For example, the α-KTx1.x subfamily (caryotoxin and Iberia toxin) uses a CSα/β scaffold, while the α-KTx6.x subfamily (HsTx1 and BmTx3) belongs to the CSα/α scaffold. Therefore, when discussing structure-function relationships, their three-dimensional folding types should be clearly distinguished to avoid scaffold classification solely based on α-KTx nomenclature.

### 2.5. Bridging Structural Diversity to Redox Outcomes

It is important to note that the structural features governing ion channel selectivity also have implications for redox biology. The electrostatic surface properties and loop flexibility of CSα/β and CSα/α scaffolds, which determine their binding specificity to Na_V_ or K_V_ channels, can indirectly influence cellular ROS generation. For instance, toxin-induced prolongation of sodium influx (via α-NaTx) or hyperpolarizing shift of activation (via β-NaTx) alters intracellular ion homeostasis, particularly Ca^2+^ levels. Elevated intracellular Ca^2+^ is a well-established trigger for NOX2 activation and mitochondrial ROS production. Thus, the same structural scaffolds that enable high-affinity ion channel recognition also position scorpion venom peptides as potential modulators of redox-sensitive signaling pathways. Differences in peptide scaffold organization may influence channel selectivity and downstream redox-related cellular responses.

**Table 1:** Major structural families and representative peptide components of scorpion venom.

Structural Family	Disulfide Pattern	Fold Type	Representative Peptides (Species)	Molecular Target/Mechanism	Citations
CSα/β (Na_V_ targeting, α-toxin)	8 Cys, 4 SS	α-helix + 3-stranded β-sheet	AaH II (*A. australis*), LqhαIT (*L. quinquestriatus*), Ts3 (*T. serrulatus*), BmKAS (*M. martensii*)	Na_V_ channel; α-toxin: site 3 binding, delayed inactivation	[15,19]
CSα/β (Na_V_-targeting, β-toxin)	8 Cys, 4 SS	α-helix + 3-stranded β-sheet	CsVII (*C. sculpturatus*), CssII (*C. suffusus*), Ts1 (*T. serrulatus*)	Na_V_ channel; β-toxin: site 4 binding, hyperpolarizing shift of activation	[19,30,43]
CSα/β (K_V_-targeting, α-KTx subfamilies 1–3)	6 Cys, 3 SS	α-helix + β-sheet (compact)	Charybdotoxin (α-KTx1.1, *L. quinquestriatus*), Iberiotoxin (α-KTx1.3, *B. tamulus*), Margatoxin (α-KTx2.2, *C. margaritatus*), Agitoxin-2 (α-KTx3.1, *L. quinquestriatus*)	K_V_ channel; pore-blocking (Lys side chain insertion into selectivity filter)	[44,45]
CSα/β (K_V_ targeting, α-KTx subfamilies 4–12)	6 Cys, 3 SS	α-helix + β-sheet (compact)	OsK-1 (α-KTx3.7, *O. scrobiculosus*), Meuk7-3 (*M. eupus*), CboK7 (α-KTx2.24, *C. bonito*)	K_V_ channel; pore-blocking or allosteric modulation	[24,46,47]
CSα/α (κ-KTx subfamily)	6 Cys, 3 SS	Two α-helices (“two-finger”)	HsTx1 (κ-KTx1.1/α-KTx6.2, *H. scorpio*), BmTx3 (α-KTx6.1, *M. martensii*), BmKK2 (*M. martensii*). Note: κ-KTx peptides belong to the α-KTx functional/sequence-based classification (subfamily 6) but adopt a CSα/α scaffold, distinct from other α-KTx subfamilies, which adopt CSα/β.	K_V_ channel: Allosteric suppression, stabilizing the non-conductive state.	[22,48,49]
CSβ (Chlorotoxin-like)	4 Cys, 2 SS	Three antiparallel β-sheets	Chlorotoxin quinquestriatus, Ce1 (*C. egyptiensis*), BmKCTa (*M. martensii*)	MMP-2, annexin A2; glioma targeting, imaging, drug delivery	[23,24]
Cysteine-free linear peptide	0 Cys, None	Amphipathic α-helix (upon membrane contact)	Pandinin-1, -2 (*P. imperator*), Pandinin-3, IsCT (*O. madagascariensis*), Hadrurin (*H. aztecus*)	Bacterial membrane disruption (carpet/barrel-stave model); antimicrobial, antifungal	[25,50,51]

**Note:** The classification in this table is based on three-dimensional folding topology (disulfide connectivity and secondary structure arrangement). The “α-KTx” designation is a functional/sequence-based classification system (based on potassium channel blocking activity and sequence homology), not a structural one. Most α-KTx subfamilies (1–3, 4–12, and others) adopt a CSα/β scaffold architecture. However, the κ-KTx subfamily (including α-KTx6.x, e.g., HsTx1 and BmTx3) adopts a CSα/α scaffold. This table separates these two structural families to avoid conflation of functional nomenclature with three-dimensional scaffold classification. All α-KTx subfamilies that adopt the CSα/β scaffold share the core architecture, with variations in loop length and surface charge distribution determining subtype selectivity. Cys: cysteine; SS: disulfide bond.

## 3. Ion Channel Modulation: Mechanisms and Selectivity

**Figure 2:**
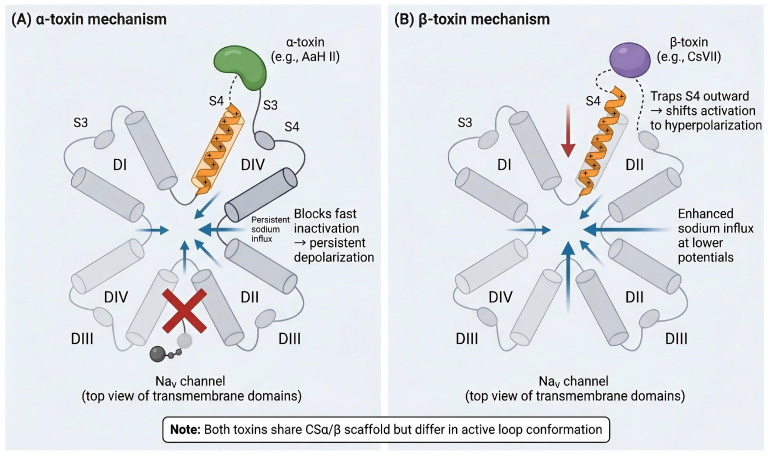
Molecular mechanisms of voltage-gated sodium channel modulation by scorpion venom peptides. This figure depicts the two major mechanisms by which scorpion toxins modulate voltage-gated sodium channels (Na_V_). α-toxins (**A**), such as AaH II, bind to receptor site 3 on domain IV of the Na_V_ channel, restricting S4 voltage-sensor movement and blocking fast inactivation; this results in persistent sodium influx and sustained membrane depolarization. β-toxins (**B**), such as CsVII, bind to receptor site 4 on domain II, trapping the voltage sensor in an activated (outward) conformation and shifting the activation threshold toward hyperpolarized potentials, thereby enhancing sodium influx at lower membrane potentials. Both toxin classes share the CSα/β scaffold but differ in their active loop conformations and surface charge distributions, which determine their distinct functional effects. Ultimately, both mechanisms increase neuronal excitability, contributing to scorpion sting pain and neurotoxicity. Refer to Section 3.1. In panel (**A**), red “X” = blockade of fast inactivation; blue arrow = persistent sodium influx. In panel (**B**), red arrow = S4 trapped in outward conformation; blue arrow = enhanced sodium influx at lower potentials. Abbreviations: Na_V_, voltage-gated sodium channel; DI–DIV, domains I–IV; CSα/β, cysteine-stabilized α-helix/β-sheet; AaH II, *Androctonus australis* Hector toxin II; CsVII, *Centruroides sculpturatus* toxin VII.

The main pharmacological value of scorpion venom peptides lies in their ability to regulate ion channels with high affinity and, in many cases, exhibit significant subtype selectivity. Voltage-gated sodium channels (Na_V_), voltage-gated potassium channels (K_V_), and transient receptor potential (TRP) channels are core regulators of electrical signal transduction in excitable tissues and are therefore major molecular targets of scorpion venom [2,15]. Studies of these peptide-channel interactions not only elucidate the toxin’s mechanism of action but also provide valuable templates for designing therapeutic ligands (Figure 2).

### 3.1. Voltage-Gated Sodium Channel Regulation Mechanism

Scorpion venom peptides are the most representative regulators of Na_V_ channels, mainly divided into α-toxins and β-toxins. Both share the CSα/β backbone, but their sites of action and gating effects differ. α-toxins primarily bind to the extracellular S3–S4 loop (receptor site 3) of the Na_V_ channel DIV domain, restricting the outward movement of the DIV voltage sensor S4, delaying rapid inactivation and prolonging channel open time, leading to sustained sodium ion influx and membrane depolarization. AaH II, LqhαIT, and Ts3 are classic examples, with Ts3 significantly reducing gating charge movement, thereby further confirming its function by restricting voltage-sensor movement [52,53]. In contrast, β-toxins primarily bind to the S3–S4 region of the DII domain (receptor site 4), stabilizing the voltage sensor in an activated conformation, shifting the channel activation curve towards hyperpolarization, and lowering the opening threshold, thereby enhancing sodium current at lower membrane potentials [43]. Although the two types of toxins have different mechanisms, both can increase neuronal excitability, which is a key molecular basis for scorpion sting pain and neurotoxicity [10].

Na_V_1.7 is the most studied subtype in pain management; loss-of-function mutations can lead to congenital analgesia, whereas gain-of-function mutations can cause hereditary pain syndromes [54]. Therefore, scorpion venom-derived Na_V_1.7 modulators are considered important lead molecules for non-opioid analgesics. OD1 can efficiently target Na_V_1.7, but also affects Na_V_1.4 and Na_V_1.6, suggesting that high selectivity between homologous isoforms remains a bottleneck in development [42,55]. Recently, the N18W variant was obtained through targeted modification of key sites in DKK2, which enhanced analgesic activity while reducing its effect on Na_V_1.4 and Na_V_1.5, demonstrating that the therapeutic window can be significantly improved through limited residue optimization based on the conserved CSα/β backbone [56].

### 3.2. Regulation of Voltage-Gated Potassium Channels

K_V_ channels are another class of targets that have been studied most extensively by scorpion venom peptides, especially the α-KTx family. K_V_ channels are another class of targets that have been studied most extensively by scorpion venom peptides, especially the α-KTx family. As discussed in Section 2.1.2, the α-KTx designation is a functional/sequence-based classification; structurally, most α-KTx peptides adopt a CSα/β scaffold, while the κ-KTx subfamily (also part of α-KTx functional classification) adopts a CSα/α scaffold. The classic “external pore blockade” mechanism described here applies primarily to CSα/β-type α-KTx peptides. Its classic mechanism is “external pore blockade”: the functional lysine side chain on the toxin’s surface is inserted into the outer vestibule of the channel and approaches the selectivity filter, thereby directly blocking K^+^ permeation. Structural studies show that this process usually does not significantly alter the overall conformation of the filter, but substituting key lysine residues significantly reduces affinity and accelerates dissociation, which is also an important reason why many scorpion venom peptides have nanomolar to picomolar potency [48,54]. In addition, not all K_V_ toxins rely on pore blockade mechanisms. Some κ-KTx peptides stabilize the non-conductive state of the channel by binding to the extracellular surface and adjacent gating domains, thereby achieving allosteric inhibition. HsTx1, a representative K_V_1.3 inhibitor, possesses picomolar activity and has become an important template for optimizing immunomodulatory peptides [48].

K_V_ subtype selectivity arises from the fine complementarity of the toxin-channel interface. The protonation state of vestibular histidine residues outside K_V_1.3 modulates margatoxin affinity, while a single amino acid substitution can significantly alter the selectivity of charybdotoxin analogs for K_V_1.2 [57,58]. CboK7 (α-KTx2.24) from Centruroides bonito also exhibits a significantly higher affinity for K_V_1.2 than for K_V_1.3 [46]. From a transformational perspective, K_V_1.3 is involved in the activation of effector memory T cells and macrophages, and is closely related to inflammation and oxidative stress. BmKK2 inhibition of K_V_1.3 can downregulate NF-κB/NLRP3 signaling, suggesting that scorpion venom peptides have anti-inflammatory and immunomodulatory potential [59].

### 3.3. Other Ion Channel Targets

Besides Na_V_ and K_V_, scorpion venom peptides can also act on many other channels. The high-conductivity calcium-activated potassium channel BK (KCa1.1) is one of the classic targets. Iberi toxin (IbTX, α-KTx1.3) inhibits K^+^ conductance by binding to the outer pore region; its potency is affected by the composition of the accessory subunits and the cellular environment. BK channels containing the β4 subunit show significantly decreased sensitivity to IbTX [60,61,62]. In terms of sensory transduction, TRPV1 is an important receptor for thermal and chemical pain. BmP01 from the East Asian pincers scorpion is the first scorpion venom peptide confirmed to directly activate TRPV1 and induce an acute pain response, suggesting that scorpion sting pain originates not only from aberrant Na_V_ activation but also involves the TRP pathway [10,63].

The acid-sensitive ion channel ASIC1a is involved in acidosis and ischemic nerve injury. Although the classic inhibitory peptide PcTx1 is derived from spider venom, its mechanism of binding to the inter-subunit interface and stabilizing a non-conductive conformation provides an important comparative model for understanding how venom peptides achieve highly selective inhibition through extracellular allosteric changes [64]. These studies indicate that the regulation of ion channels by venom peptides is far more than a single-pore-blocking mode; it encompasses multiple mechanisms, including gating capture, allosteric stabilization, and auxiliary subunit-dependent regulation.

### 3.4. Integrated Determinants of Selectivity

The high selectivity of scorpion venom peptides stems from the synergistic effect of multiple structural factors. First, surface electrostatic complementarity may influence initial recognition efficiency. Most Na_V_-active toxins are strongly alkaline, which is more conducive to binding to negatively charged voltage-sensing regions, whereas K_V_ toxins typically have a more balanced charge distribution, making them more suitable for recognition in the outer pore region [65]. Second, the local flexibility of the exposed loop region endows the toxin with induced-fit ability, enabling it to adapt to highly homologous but subdivided channel subtypes [66]. The membrane lipid environment can influence the orientation, accumulation, and contact efficiency of toxins on the membrane surface, especially for gated toxins [67].

On balance, the CSα/β and CSα/α scorpion venom peptide scaffolds demonstrate how a finite structural framework can evolve into diverse recognition strategies. In the future, combining cryo-electron microscopy, electrophysiology, computational design, and directed evolution technologies will further promote the transformation of scorpion venom peptides from molecular probes into highly selective analgesics, immunomodulators, and anti-inflammatory drug candidates.

### 3.5. Translational Safety Considerations: Subtype Selectivity, Cross-Reactivity Risks, and Validation Requirements

For venom-derived peptides to advance as therapeutics, subtype selectivity is a critical safety determinant. The high structural homology among voltage-gated ion channel subtypes presents a major challenge for venom-derived peptide therapeutics, as cross-reactivity with off-target channels can lead to significant adverse effects.

#### 3.5.1. Selectivity Panels Required for Therapeutic Development

Before clinical development, lead peptide candidates should be subjected to selectivity panels that include all major Na_V_ isoforms (Na_V_1.1–Na_V_1.9, with emphasis on Na_V_1.4, Na_V_1.5, Na_V_1.6, and Na_V_1.7) and K_V_ isoforms (K_V_1.1–K_V_1.6, with emphasis on K_V_1.3 for immunomodulatory indications). Automated patch-clamp platforms (e.g., QPatch and Qube) have become essential tools for high-throughput electrophysiological profiling of venom-derived peptides against channel panels [68].

#### 3.5.2. K_V_1.3 Immune Relevance Versus K_V_1.2/K_V_1.1 Neurological Relevance

A critical selectivity consideration for immunomodulatory peptides is the balance between K_V_1.3 blockade (desired for suppressing effector memory T cells in autoimmune diseases) and off-target activity at K_V_1.1 and K_V_1.2 (which can cause neurological adverse effects). For example, the selective K_V_1.3 blocker J123 exhibits > 1000-fold selectivity over K_V_1.1 and ~30-fold over K_V_1.2, demonstrating that such selectivity is achievable through rational design [69]. Similarly, Vm24 shows picomolar affinity for K_V_1.3 (Kd ~2.9 pM) with excellent selectivity over other K_V_ channels [70].

#### 3.5.3. Need for Electrophysiological Validation Across Homologous Channels

Computational prediction of cross-reactivity, while valuable, is insufficient for safety assessment. Empirical electrophysiological validation using patch-clamp techniques remains the gold standard. Studies have shown that even single amino acid substitutions can dramatically alter selectivity profiles. For instance, the BeM9GG derivative of scorpion α-neurotoxin selectively lost activity against cardiac Na_V_1.5 while retaining activity against other isoforms, demonstrating that structure-guided optimization can mitigate cardiac risks [71]. However, such engineering requires systematic electrophysiological screening across all potentially cross-reactive channels. In essence, thorough selectivity profiling combining computational prediction, high-throughput automated patch-clamp screening, and confirmatory manual patch-clamp electrophysiology is essential for de-risking venom-derived peptide therapeutics prior to clinical development.

### 3.6. Linking Ion Channel Modulation to ROS and Inflammation: Context-Dependent Relationships

Beyond direct safety considerations, ion channel modulation by scorpion venom peptides can influence cellular ROS production, but this relationship is not automatic or universal. Whether a given toxin increases or decreases ROS depends on several contextual factors that should be explicitly considered.

Cell type matters. In excitable cells such as neurons and cardiomyocytes, toxin-induced Na^+^ influx or K^+^ efflux directly alters membrane potential and Ca^2+^ homeostasis. In contrast, in non-excitable cells such as microglia or immune cells, the same toxin may have different effects because these cells rely less on voltage-gated channels for basal activity. Most studies linking scorpion toxins to ROS generation have been conducted in microglia or neurons; generalization to other cell types requires experimental validation. Channel subtype may contribute to the direction of effect. Different Na_V_ or K_V_ isoforms have distinct tissue distributions and gating properties. For example, α-NaTx-mediated prolongation of Na^+^ influx through Na_V_1.7 in sensory neurons may increase intracellular Ca^2+^ via reverse Na^+^/Ca^2+^ exchange, activating NOX2. However, the same toxin acting on Na_V_1.5 in cardiomyocytes may produce different Ca^2+^ dynamics and ROS outcomes. Similarly, blockade of K_V_1.3 in T cells suppresses activation and may reduce inflammation-associated ROS, whereas blockade of K_V_1.2 in neurons may prolong action potentials and increase Ca^2+^-dependent ROS. Toxin concentration and exposure duration critically determine outcomes. At low nanomolar concentrations, many scorpion toxins selectively modulate channel gating without causing overt cell stress. At higher micromolar concentrations, non-specific effects or excessive ion flux may trigger mitochondrial dysfunction and ROS burst. Acute exposure (minutes to hours) typically produces reversible changes, whereas chronic exposure (days to weeks) may induce adaptive responses such as Nrf2 upregulation. Most published studies use short-term exposure (hours) at moderate concentrations (10–100 nM); the long-term effects remain poorly characterized. Pro-oxidant versus antioxidant outcomes depend on context. In some experimental settings, increased ROS following toxin exposure triggers Nrf2-mediated antioxidant gene expression as a compensatory response. Thus, the same toxin can be described as both pro-oxidant (increasing ROS) and antioxidant (upregulating defenses) depending on the timing and endpoint measured. This dual effect should not be conflated with the cancer-selective pro-oxidant activity discussed in Section 4.5.5, which operates through a distinct mechanism (mitochondrial targeting).

At its core, ion channel modulation by scorpion venom peptides can lead to ROS generation and inflammatory signaling under specific conditions, particularly in excitable cells following sustained Na^+^ influx or K_V_ blockade. However, this relationship is context-dependent and should not be assumed to occur in all cell types, at all concentrations, or with all toxin subtypes. Understanding these contextual determinants is essential for interpreting both the therapeutic potential and toxicity profiles of scorpion venom peptides.

### 3.7. Toxicity Profiles and Dose-Dependent Adverse Effects of Scorpion Venom Peptides

Important note on data comparability: The quantitative toxicity parameters (LD50, TD50, ED50, and therapeutic index) reported in this section are derived from different published studies that used varying experimental conditions, including mouse strains (e.g., BALB/c, C57BL/6, ICR), administration routes (intraperitoneal, intravenous, subcutaneous, intracerebroventricular), peptide purity (crude venom vs. HPLC-purified peptide), and endpoint definitions (e.g., death within 24 h vs. 72 h, specific behavioral signs). Therefore, direct cross-comparison of absolute values between different studies should be made with caution. The values presented below are provided to illustrate general potency ranges and relative trends, not as absolute benchmarks that can be compared across different experimental setups. Readers are encouraged to consult the original publications for complete experimental details. Understanding the toxicity profile of scorpion venom and its peptide components is critical for evaluating their therapeutic potential and safety margin. Scorpion venom toxicity is primarily mediated by neurotoxins that target voltage-gated ion channels, but the dose-dependent adverse effects vary significantly between whole venom and purified peptides.

#### 3.7.1. Whole Venom Toxicity

Scorpion envenomation produces a spectrum of clinical manifestations ranging from local pain and inflammation to severe systemic toxicity. The lethality of scorpion venom is typically quantified by the median lethal dose (LD50) in animal models. For *Leiurus quinquestriatus* (the deathstalker scorpion), the intraperitoneal LD50 in adult mice ranges from approximately 0.25 to 0.50 mg/kg, with death occurring within 24–48 h as the standard endpoint [10]. For *Androctonus australis*, the intraperitoneal LD50 in mice ranges from 0.32 to 0.75 mg/kg [10,72]. Because these values come from different studies with potentially different protocols (mouse strain, venom source, purity, and observation period), they should not be directly compared as absolute measures. Systemic envenomation symptoms include autonomic storms (hypertension, tachycardia, bradycardia and hyperthermia), cardiotoxicity (myocarditis, arrhythmias and pulmonary edema), neurotoxicity (seizures and neuromuscular paralysis), and local tissue damage (edema, hemorrhage and necrosis) [8,10].

#### 3.7.2. Dose-Dependent Adverse Effects of Purified Peptides

Purified scorpion venom peptides exhibit dose-dependent toxicity profiles. The therapeutic window is defined by the ratio between the effective dose and the toxic or lethal dose. (i) Cardiotoxicity: Several scorpion toxins active on Na_V_ channels have demonstrated dose-dependent cardiotoxic effects. At low (nanomolar) concentrations, they can prolong action potential duration and enhance contractility, while at higher (micromolar) concentrations, they may induce arrhythmias, contracture, and cardiac failure. The β-toxin CsVII, for example, shows cardiac effects at higher doses, while its analgesic effects are observed at lower doses, suggesting a narrow therapeutic window under the specific experimental conditions tested [19]. (ii) Neurotoxicity: At sub-lethal doses, scorpion neurotoxins cause hyperexcitability, pain, and autonomic dysfunction. At higher doses, they can induce seizures, respiratory distress, and paralysis. The toxicity profile of α-toxins is exemplified by AaH II, a well-studied toxin from *Androctonus australis*. AaH II produces neurological symptoms at low doses and is lethal at higher doses, with a dose ratio (lethal/neurotoxic) indicative of a narrow therapeutic margin. It must be noted that these values come from different studies with varying experimental conditions (mouse strain, administration route), and direct cross-comparison of absolute numbers is not scientifically valid. Instead, these data illustrate the general principle that increasing doses lead to more severe neurotoxic outcomes [10,73].

#### 3.7.3. Selectivity and Safety Margins in Therapeutic Candidates

For venom-derived peptide therapeutics, the goal is to maximize selectivity for the target channel while minimizing off-target toxicity. Engineered peptides have shown improved safety margins. For example, the engineered Na_V_1.7-selective variant DKK2-N18W showed no observable adverse effects at doses substantially higher than its analgesic effective dose in a mouse study, indicating an improved safety margin compared to the native toxin [56]. Similarly, the selective K_V_1.3 blocker Vm24 demonstrated immunosuppressive activity at doses well below those causing observable toxicity in mice [70].

#### 3.7.4. Implications for Therapeutic Development

The dose-dependent toxicity profiles discussed above have several implications for developing scorpion venom peptides into therapeutics: (i) thorough preclinical safety pharmacology studies, including cardiovascular and CNS safety assessments, are required before clinical trials; (ii) therapeutic windows must be established through rigorous dose–response studies; (iii) lead optimization should prioritize engineering strategies that enhance selectivity and reduce off-target toxicity (e.g., cyclization, D-amino acid substitution, or Fc fusion); and (iv) clinical development should start with conservative dose escalation protocols to identify the maximum tolerated dose and dose-limiting toxicities in humans.

Briefly, while scorpion venom peptides possess potent pharmacological activities, their dose-dependent toxicity profiles, particularly cardiotoxicity and neurotoxicity, must be carefully addressed during therapeutic development. The narrow therapeutic windows of many natural toxins highlight the importance of engineering strategies to improve selectivity and safety margins [8,10,72].

Caveats for interpreting toxicity data across studies: The quantitative values summarized above serve to illustrate general potency relationships and safety margins for individual peptides within their respective studies. However, several factors limit cross-study comparisons: (i) animal strain differences: BALB/c, C57BL/6, ICR, and CD-1 mice can show different sensitivities to the same toxin; (ii) administration route: intravenous injection typically produces higher bioavailability and lower LD50 values compared to intraperitoneal, subcutaneous, or intracerebroventricular routes; (iii) peptide purity: crude venom contains multiple components that may synergistically enhance or antagonize toxicity, whereas purified peptides generally show cleaner dose–response relationships; (iv) endpoint definitions: studies may define toxicity based on different criteria (e.g., respiratory distress, seizure onset, or death within 24 vs. 72 h). Therefore, readers are advised to consult the original literature for detailed experimental protocols rather than relying on isolated numeric values when comparing peptides across different studies.

## 4. Beyond Ion Channels: Emerging Targets and Pharmacological Profiles

**Figure 3:**
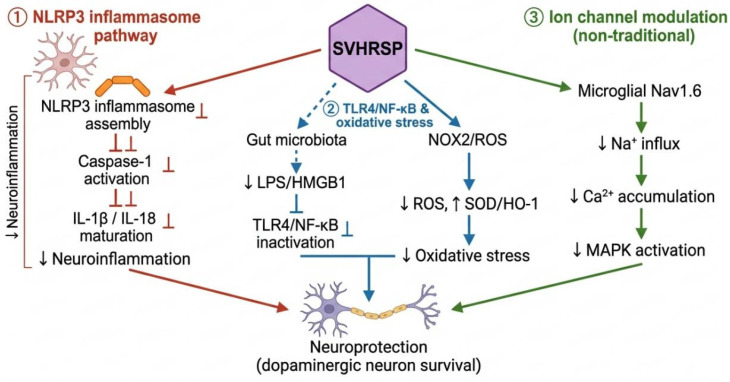
Multi-target neuroprotective mechanism of SVHRSP. This figure illustrates the three interconnected pathways through which SVHRSP (scorpion venom heat-resistant synthetic peptide) exerts neuroprotection. First, SVHRSP inhibits NLRP3 inflammasome assembly and caspase-1 activation in microglia, reducing IL-1β/IL-18 maturation and neuroinflammation. Second, SVHRSP reshapes gut microbiota composition, lowering circulating and cerebral LPS/HMGB1 levels, which in turn limits TLR4/NF-κB activation and suppresses oxidative stress. Third, SVHRSP reduces microglial Na_V_1.6 expression, decreasing sodium influx, intracellular Ca^2+^ accumulation, and MAPK pathway activation. The convergence of these anti-inflammatory, antioxidant, and ion channel-modulating pathways leads to reduced oxidative stress, dampened neuroinflammation, and enhanced dopaminergic neuron survival. Refer to Section 4.4. Abbreviations: SVHRSP, scorpion venom heat-resistant synthetic peptide; NLRP3, NLR family pyrin domain containing 3; IL-1β/IL-18, interleukins-1β and -18; TLR4, Toll-like receptor 4; NF-κB, nuclear factor-kappa B; LPS, lipopolysaccharide; HMGB1, high-mobility group box 1; Na_V_1.6, voltage-gated sodium channel subtype 1.6; MAPK, mitogen-activated protein kinase. Red solid arrows: Regulatory pathways by which SVHRSP exerts neuroprotective effects through inhibition of the NLRP3 inflammasome pathway. Blue solid arrows: Two branch pathways by which SVHRSP exerts neuroprotective effects through regulation of the gut microbiota-oxidative stress axis. Blue dashed arrows: Indirect regulation of the gut microbiota by SVHRSP. Green solid arrows: Flow of SVHRSP’s neuroprotective effects through non-traditional ion channel regulation mechanisms. “↓”: The molecule/process is inhibited, reduced, or decreased. “↑”: The molecule/process is activated, increased, or enhanced. “⊥”: The molecule/process is directly blocked or inhibited.

Although scorpion venom peptides were initially characterized as selective ion channel modulators, accumulating evidence has revealed that their pharmacological spectrum extends far beyond membrane excitability. An emerging and particularly relevant theme is their ability to regulate oxidative stress, neuroinflammation, and mitochondrial function—processes that are intimately linked to ion channel activity. As discussed in Section 3, toxin-induced changes in Na^+^ and Ca^2+^ homeostasis can trigger ROS generation and activate inflammatory cascades. Conversely, scorpion venom peptides have been shown to directly engage antioxidant pathways, including Nrf2-ARE transcriptional activation and NOX2 inhibition, independent of or parallel to their ion channel effects. Recent studies have demonstrated that scorpion venom peptides, particularly thermostable derivatives from *Mesobuthus martensii*, exhibit protective effects in experimental models of Parkinson’s disease, Alzheimer’s disease, and epilepsy. This raises the possibility that scorpion venom peptides can simultaneously target ion channels, oxidative stress pathways, mitochondrial homeostasis, and glial cell signaling networks, positioning them as multi-functional therapeutic candidates for complex neurological disorders [72,74,75] (Figure 3).

### 4.1. Analgesic Mechanisms and Targets

Scorpion venom remains an important natural resource for the development of novel analgesics. Na_V_1.7, Na_V_1.8, and Na_V_1.9 are closely associated with congenital analgesia, erythromelalgia, and paroxysmal pain syndrome, thereby making them key targets for non-opioid analgesics. Active peptides in scorpion venom and other arachnid venoms provide abundant templates for the design of highly selective analgesic ligands [76]. Mechanistically, α-NaTx prolongs channel opening time by delaying inactivation, while β-NaTx lowers the activation threshold; both enhance the excitability of nociceptive neurons and pain transmission [10]. Current research has shifted from broad-spectrum Na_V_ regulation to optimizing Na_V_1.7 subtype selectivity, aiming to reduce off-target risks to the heart and skeletal muscle while maintaining analgesic activity [55]. Structurally, most analgesic-related scorpion venom peptides retain the CSα/β backbone, while surface charge distribution and active ring conformation determine their subtype preference.

### 4.2. Anticancer Mechanisms and Targets

Scorpion venom peptides exert anticancer effects through multiple distinct mechanisms, which can be classified into five categories: direct cytotoxicity, tumor-homing/targeting, imaging, drug delivery, and immunomodulation. The following subsections address each category separately, as they have different translational applications and mechanisms of action.

#### 4.2.1. Direct Cytotoxic Peptides

Several scorpion venom peptides directly kill cancer cells through membrane disruption, apoptosis induction, or mitochondrial dysfunction. For example, Smp24 induces mitochondrial apoptosis in HepG2 cells and inhibits tumor growth in vivo [77]. The scorpion venom peptide S6540, isolated from *Tityus serrulatus*, targets mitochondria in A549 non-small cell lung cancer cells, induces excessive ROS generation, triggers PI3K/Akt inactivation, and leads to caspase-independent apoptosis [78]. Androcin 18-1, an 18-amino acid peptide from *Androctonus crassicauda* venom, exerts anti-glioblastoma activity against U87 cells by inducing mitochondrial dysfunction and ROS accumulation [79]. These direct cytotoxic peptides are candidates for stand-alone anticancer therapy, though their selectivity for cancer versus normal cells requires further optimization.

#### 4.2.2. Tumor-Homing/Targeting Peptides-Chlorotoxin

Chlorotoxin is one of the most representative molecules in scorpion venom antitumor research. This CSβ family peptide is rich in β-sheet content and can recognize glioma-related molecules, such as matrix metalloproteinase-2 (MMP-2) and annexin A2, with high affinity [23,72]. After binding, it induces complex internalization, disrupts Ca^2+^ homeostasis, and remodels the cytoskeleton, thereby inhibiting tumor cell migration and invasion [80]. However, it is important to note that chlorotoxin’s primary translational value is as a tumor-targeting ligand rather than as a broadly cytotoxic anticancer drug. Its intrinsic cytotoxicity is modest, and its main utility lies in its ability to selectively deliver therapeutic or diagnostic payloads to glioma cells [23].

#### 4.2.3. Imaging Agents

Taking advantage of chlorotoxin’s tumor-homing properties, the synthetic analog TM-601 has been developed for glioma imaging. When conjugated to fluorophores (e.g., indocyanine green) or radionuclides (e.g., ^131^I), chlorotoxin enables fluorescence-guided surgery and multimodal imaging of brain tumors. Early phase I/II clinical trials using intracavitary injection of ^131^I-TM-601 demonstrated good tolerability and tumor-selective retention in patients with recurrent high-grade gliomas [81]. This imaging application represents one of the most clinically advanced uses of a scorpion venom-derived peptide.

#### 4.2.4. Drug Delivery Ligands

Chlorotoxin has been widely used to functionalize nanocarriers for targeted drug delivery to brain tumors. Chlorotoxin-decorated nanoparticles improve blood–brain barrier penetration and enhance the accumulation of therapeutic payloads (e.g., chemotherapeutics and siRNA) at tumor sites [23]. Beyond nanoparticle-based systems, chlorotoxin has been incorporated into exosomes and chimeric antigen receptor T cell (CAR-T) constructs for glioma-targeted therapy. These delivery platforms exploit chlorotoxin’s binding specificity without relying on its intrinsic cytotoxicity.

#### 4.2.5. Immuneomodulatory Venom Components

Beyond direct tumor cell targeting, some scorpion venom components exert anticancer effects by remodeling the tumor immune microenvironment. For example, the F1 component of the European scorpion (Mesobuthus eupeus) can transform IL-4-polarized M2 macrophages into an M1-like phenotype, and the conditioned medium of treated macrophages can inhibit the proliferation and migration of CT-26 colon cancer cells [8,75]. One interpretation is that certain scorpion venom peptides may have indirect antitumor activity through immunomodulation, representing a distinct mechanism from direct cytotoxicity or tumor targeting.

Briefly, scorpion venom peptides offer a diverse toolbox for cancer-related applications, ranging from direct cytotoxic agents (e.g., Smp24 and S6540) to tumor-homing ligands (chlorotoxin), imaging probes (TM-601), drug delivery vehicles (chlorotoxin-decorated nanoparticles), and immunomodulatory components (F1 fraction). Recent reviews have highlighted oncology as one of the most promising translational directions for scorpion venom pharmacology [76].

### 4.3. Antimicrobial Activity

Scorpion venom contains a large number of non-disulfide-linked antimicrobial peptides, which are increasingly seen as alternatives to traditional antibiotics. Many of these peptides possess broad-spectrum antimicrobial activity and may exert less selective pressure for resistance than classic small-molecule drugs. Their emergence has greatly expanded the pharmacological properties of scorpion venom beyond ion channel regulation [2,8]. A systematic review published in 2022 summarized the structural diversity of scorpion venom peptides with antimicrobial, antifungal, and antiparasitic properties and concluded that these molecules are valuable templates for the design of next-generation anti-infective drugs [82]. Most antimicrobial scorpion venom peptides are linear, cysteine-free molecules, such as the pandinine family. Unlike disulfide-rich neurotoxins, these peptides typically form amphiphilic α-helices upon contact with cell membranes and disrupt microbial envelopes through carpet-like or barrel-like mechanisms. Pandinin 1 and Pandinin 2 have been reported to increase bacterial membrane permeability and induce intracellular leakage [15,50,77]. Recent reviews further indicate that scorpion venom peptides may also inhibit fungi and certain viruses, highlighting their potential as multifunctional anti-infective agents [8].

### 4.4. Neuroprotective Effects

**Figure 4:**
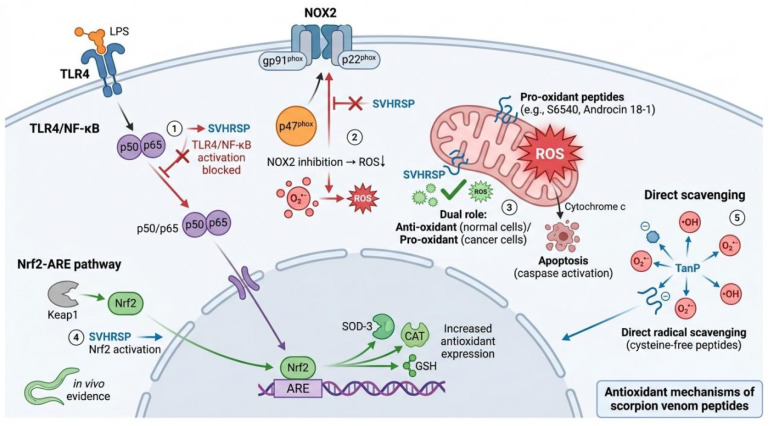
Schematic diagram of the antioxidant mechanisms of scorpion venom peptides. This figure summarizes the multi-pathway antioxidant activities of scorpion venom peptides, centered on SVHRSP. SVHRSP activates the Nrf2-ARE pathway by promoting Nrf2 release from Keap1 and its nuclear translocation, upregulating SOD-3, CAT, and GSH expression (validated in *C. elegans*). SVHRSP also directly inhibits NOX2 by blocking p47^phox^ membrane translocation, reducing ROS generation at its source; siRNA knockdown of NOX2 abolishes its protective effects. Additionally, cysteine-free linear peptides (e.g., TanP) directly scavenge free radicals independent of signaling pathways. In cancer cells, certain peptides (e.g., S6540, Androcin 18-1) induce mitochondrial ROS overproduction and apoptosis, demonstrating a therapeutically relevant dual pro-oxidant/antioxidant selectivity. Refer to Section 4.5. Abbreviations: SVHRSP, scorpion venom heat-resistant synthetic peptide; Nrf2, nuclear factor erythroid 2-related factor 2; ARE, antioxidant response element; Keap1, Kelch-like ECH-associated protein 1; SOD-3, superoxide dismutase-3; CAT, catalase; GSH, glutathione; NOX2, NADPH oxidase 2; p47^phox^, cytosolic subunit of NOX2; ROS, reactive oxygen species; *C. elegans*, *Caenorhabditis elegans*.

Among recently identified scorpion venom-derived molecules, SVHRSP has emerged as a representative multi-target neuroprotective peptide. Current evidence suggests its beneficial effects primarily manifest in three interrelated pathways (Figure 3). First, SVHRSP inhibits NLRP3 inflammasome-mediated neuroinflammation in microglia. Studies by Zhang et al. have shown that this peptide inhibits inflammasome assembly and caspase-1 activation, thereby reducing the maturation and release of IL-1β and related cytokines. Pharmacological inhibition or gene knockout of NLRP3 significantly attenuates this protective effect, indicating that this pathway is a major downstream target [83]. In these studies, SVHRSP was administered at doses of 1–10 mg/kg (intraperitoneally or intravenously) in rodent models, or at concentrations of 0.1–10 μM in microglial cell cultures. A review on venom peptides regulating the NLRP3 inflammasome also highlights the anti-inflammatory potential of scorpion venom peptides [84]. Second, SVHRSP attenuates oxidative stress and the TLR4/NF-κB signaling pathway. This peptide has been reported to remodel gut microbiota composition and reduce circulating and intracranial LPS/HMGB1 levels, thereby limiting activation of the TLR4/NF-κB pathway in the brain [85]. Simultaneously, it can inhibit NOX2 assembly, reduce reactive oxygen species production, and enhance the activity of endogenous antioxidant enzymes [86,87]. SVHRSP may regulate unconventional ion channel targets in glial cells. Studies have shown that it can reduce Na_V_1.6 expression in microglia, inhibit sodium current and intracellular Ca^2+^ accumulation, and attenuate activation of the MAPK pathway [88]. These findings support SVHRSP as a multi-mechanism neuroprotective lead compound and elucidate how scorpion peptides can be applied to the treatment of complex neurological diseases (Figure 4).

While SVHRSP suggests promising multi-target neuroprotective activity in preclinical models, several important limitations should be acknowledged to provide a balanced perspective. First, the majority of SVHRSP-related findings have been reported by a single research group [74,83,85,88,89,90]. Independent replication by other laboratories is needed to confirm the reproducibility and generalizability of these observations. Second, systematic dose–response studies are limited. Most studies have used a narrow range of SVHRSP concentrations (typically 0.1–10 μM in vitro and 1–10 mg/kg in vivo), and the therapeutic window (the ratio between efficacious and toxic doses) has not been clearly established. Third, pharmacokinetic data are incomplete. Although SVHRSP has been detected in cerebrospinal fluid after systemic administration [91], quantitative parameters such as plasma half-life, bioavailability, volume of distribution, and brain-to-plasma ratio have not been systematically reported. Fourth, blood–brain barrier (BBB) penetration has been demonstrated qualitatively but not quantified. The exact mechanism of BBB transport (passive diffusion vs. receptor-mediated transcytosis) remains unknown. Fifth, comprehensive toxicity, immunogenicity, and long-term safety studies are absent. No published data are available on chronic toxicity, off-target effects, anti-drug antibody formation, or organ-specific accumulation following repeated administration. Sixth, and most Interestingly, no human clinical evidence currently exists for SVHRSP, and no human clinical efficacy data have yet been published for this peptide. SVHRSP has received Investigational New Drug (IND) approval from China’s NMPA as a Class 1.1 innovative drug for clinical evaluation in epilepsy. This regulatory milestone permits phase I clinical trials to assess safety, tolerability, and pharmacokinetics in humans but does not yet constitute evidence of therapeutic efficacy. Clinical proof-of-concept in patients with Parkinson’s disease, Alzheimer’s disease, or epilepsy remains to be established. Together, while SVHRSP is a mechanistically well-characterized and promising lead candidate, substantial work is needed to address these limitations before it can be considered a clinically validated therapeutic.

### 4.5. Antioxidant Mechanism of Scorpion Venom Peptides

In recent years, the antioxidant activity of scorpion venom peptides has evolved from an ancillary observation of ion channel modulation to an independent and mechanistically distinct research direction. Critically, the pathways described below—Nrf2-ARE activation, NOX2 inhibition, and direct radical scavenging—are not isolated from the ion channel effects discussed in Section 3; rather, they represent parallel or downstream mechanisms that converge on redox homeostasis. For example, NOX2 is directly activated by elevated intracellular Ca^2+^ following Na_V_ channel modulation, while Nrf2 activation can be triggered by both ROS and Ca^2+^ signals. Thus, the antioxidant activities of scorpion venom peptides complement their ion channel-modulating properties, offering multi-tiered protection against oxidative injury. Existing evidence suggests that scorpion venom peptides exert their antioxidant effects through multiple pathways, including activating the Nrf2-ARE signaling axis, targeting and inhibiting NADPH oxidase (NOX2), regulating the endogenous antioxidant enzyme system, and directly scavenging free radicals, forming a multi-level, multi-target oxidative stress regulatory network [85].

It is important to distinguish between direct antioxidant effects and indirect redox-modulatory or cytoprotective effects. Direct effects include chemical radical scavenging (where a peptide directly neutralizes ROS) and direct enzyme inhibition (where a peptide binds and inhibits a ROS-generating enzyme). Indirect effects include transcriptional activation of antioxidant genes (Nrf2-ARE), upregulation of endogenous antioxidant enzymes (SOD, CAT, GSH), and suppression of pro-inflammatory signaling that secondarily reduces oxidative stress. The following subsections explicitly label each mechanism as direct or indirect.

#### 4.5.1. Transcriptional Activation of the Nrf2-ARE Pathway

In the Nrf2-ARE pathway, the representative synthetic peptide SVHRSP alleviated cognitive impairment in an Alzheimer’s disease model exacerbated by PM2.5 exposure by upregulating Nrf2 expression and inhibiting endoplasmic reticulum stress and neuronal pyroptosis. This is an indirect cytoprotective mechanism because it requires transcription, translation, and protein synthesis. SVHRSP does not directly scavenge radicals; instead, it promotes Nrf2 nuclear translocation and ARE-driven gene expression. SVHRSP can also alleviate PM2.5 exposure-induced neuronal necrosis and apoptosis by regulating the long non-coding RNA Gm6410 [90]. Furthermore, using a *Caenorhabditis elegans* model, SVHRSP was shown to reduce ROS levels and increase SOD-3 activity. Gene expression analysis revealed that SVHRSP upregulated multiple genes with distinct biological functions: sod-3 (superoxide dismutase) and ctl-1 (catalase) are directly involved in antioxidant defense; egl-1 (a BH3-only protein) is primarily associated with apoptosis regulation; and cat-2 (tyrosine hydroxylase) is a key enzyme in dopamine biosynthesis. While the upregulation of sod-3 and ctl-1 directly supports the antioxidant capacity of SVHRSP, the increased expression of egl-1 and cat-2 may reflect stress-induced compensatory responses or pleiotropic effects of the peptide on multiple signaling pathways. Together, these findings validate the ability of scorpion venom peptides to activate endogenous defense systems across different species, while also highlighting the functional diversity of the transcriptional response [89]. These findings indicate that scorpion venom peptides can establish a systemic defense against oxidative stress at the transcriptional level.

#### 4.5.2. Targeted Inhibition of NOX2

In terms of NADPH oxidase targeting inhibition, SVHRSP demonstrated precise regulation of NOX2 in a Parkinson’s disease model. NOX2 inhibition is an indirect antioxidant mechanism because it blocks the production of new ROS rather than scavenging existing ROS. SVHRSP prevents p47^phox^ membrane translocation, thereby reducing superoxide generation at its source. Mechanistic studies showed that SVHRSP reduces reactive oxygen species (ROS) generation at its source by blocking the translocation of the cytoplasmic subunit p47^phox^ to the cell membrane, thereby inhibiting the assembly and activation of the NOX2 holoenzyme. Knockdown of NOX2 expression via siRNA significantly weakened the inhibitory effects of SVHRSP on LPS- and rotenone-induced pro-inflammatory cytokine gene expression and related neurotoxicity, validating NOX2 as its core target [74]. It should be noted that this evidence suggests pathway-level inhibition of NOX2 assembly. Direct measurement of NOX2 enzymatic activity in cell-free systems has not yet been reported.

#### 4.5.3. Regulation of Endogenous Antioxidant Enzyme Systems

Scorpion venom peptides can also regulate the activity of endogenous antioxidant enzyme systems. Increased SOD, CAT, and GSH levels reflect an indirect adaptive cellular response, not direct radical scavenging by the peptide itself. Smp24 appears to enhance the cell’s own antioxidant capacity through mechanisms that remain to be fully elucidated. Smp24 not only inhibited tumor growth in a mouse model of solid Ehrlich ascites carcinoma but also significantly increased the levels of superoxide dismutase (SOD), catalase (CAT), and glutathione (GSH), while decreasing malondialdehyde (MDA) and nitric oxide (NO) levels [77]. This simultaneous regulation of multiple antioxidant enzymes suggests that scorpion venom peptides can systematically enhance the organism’s antioxidant defense capacity, rather than acting on a single target.

#### 4.5.4. Direct Free Radical Scavenging

Some linear anionic peptides (such as TanP) can directly scavenge free radicals, and their effects are independent of intracellular signaling pathways, providing new insights for developing structurally concise antioxidant candidate molecules [92]. This is the only direct antioxidant mechanism among those described in this section. TanP and related cysteine-free linear peptides chemically neutralize free radicals without requiring cells, enzymes, or signaling pathways. This mechanism is analogous to that of plant-derived polyphenols. These cysteine-free linear peptides are structurally distinct from classical disulfide-rich toxins, and their antioxidant mechanism more closely resembles that of plant-derived polyphenols or flavonoids, offering advantages such as simple structure and ease of synthesis.

#### 4.5.5. Dual Pro-Oxidant/Antioxidant Selectivity

In cancer cells, S6540 and Androcin 18-1 exert a direct pro-oxidant effect by targeting mitochondria and inducing ROS overproduction. In normal cells, by contrast, the same peptides tend to suppress oxidative stress through indirect mechanisms (e.g., pathway modulation), reflecting differences in membrane potential, metabolic state, and surface charge distribution between normal and cancerous cells. It is noteworthy that the relationship between scorpion venom peptides and reactive oxygen species is not a one-way “inhibition.” In tumor cells, some scorpion venom peptides (such as S6540 and Androcin 18-1) induce apoptosis by promoting excessive mitochondrial ROS production, exhibiting dual selectivity for “pro-oxidation-antioxidation.” The scorpion venom peptide S6540 targets mitochondria, induces ROS overproduction, followed by PI3K/Akt inactivation and caspase-independent apoptosis [78]. Androcin 18-1 similarly exerts anti-glioblastoma activity by inducing mitochondrial dysfunction and ROS accumulation [79]. This difference may stem from differences in membrane potential, metabolic state, and surface charge distribution between normal and tumor cells, providing a theoretical basis for the targeted application of scorpion venom peptides in tumor therapy [74,93].

#### 4.5.6. Summary of Antioxidant Mechanisms

The antioxidant-related activities of scorpion venom peptides can be classified into direct and indirect mechanisms. The only direct mechanism documented to date is free radical scavenging by certain cysteine-free linear peptides (e.g., TanP), which chemically neutralize ROS without requiring cellular machinery. Indirect mechanisms include: (i) transcriptional activation of the Nrf2-ARE pathway, which may influence endogenous antioxidant enzymes; (ii) targeted inhibition of NOX2, which suppresses ROS generation at its source; (iii) regulation of endogenous antioxidant enzyme levels (SOD, CAT, GSH); and (iv) in cancer cells, direct mitochondrial ROS induction (pro-oxidant), while the same peptides exert indirect antioxidant effects in normal cells. Elucidating this distinction is critical for interpreting experimental data and for designing peptide-based therapeutics with desired redox-modulatory profiles (Table 2).

## 5. Peptide Engineering and Therapeutic Development

### 5.1. Molecular Optimization and Delivery Strategies

Despite the high affinity and subtype selectivity of natural scorpion venom peptides for ion channel targets, their development as therapeutics has long been hampered by three major bottlenecks: rapid proteolytic degradation, potential immunogenicity, and limited tissue penetration, particularly the difficulty in crossing the blood–brain barrier (BBB). Recent advances in peptide engineering, drug delivery, and synthetic biotechnology have begun to address these obstacles (Table 3).

Improving metabolic stability is a primary goal. Cyclic skeletalization and terminal modification are commonly used methods. Cyclic analogs of BmKTX significantly prolonged resistance to enzymatic degradation while maintaining potent K_V_1.3 inhibitory activity (half-life extended from <30 min to >4 h in plasma), demonstrating that conformational restriction can maintain biological activity while reducing protease sensitivity [94]. Introducing non-natural amino acids provides a complementary strategy. Replacing key residues of scorpion venom with D-amino acid analogs significantly improved its plasma stability in non-human primates and prolonged its in vivo half-life (half-life increased from ~2 h to >12 h), while maintaining immunosuppressive efficacy [95]. Half-life extension can also be achieved through fusion engineering. Integrating engineered scorpion venom peptide motifs into Fc-based multivalent constructs yielded a selective K_V_1.3 inhibitor with prolonged systemic exposure (half-life ~5 days in cynomolgus monkeys) and sustained inhibition of IL-2 secretion in cynomolgus monkey T cells (>80% suppression for 7 days), supporting the platform’s potential for treating autoimmune diseases [45].

Delivery innovation is particularly important for central nervous system applications. Chlorine-functionalized nanoparticles have attracted significant attention for glioblastoma treatment because chlorine can promote receptor-mediated trans-blood-brain-barrier transport while maintaining tumor-targeting specificity (tumor-to-brain ratio > 10:1 at 24 h post-injection) [96]. Physical modulation of the BBB is another option: focused ultrasound combined with microbubbles can transiently and reversibly increase BBB permeability by 2- to 10-fold in specific brain regions, enhancing peptide delivery by up to 50-fold [97]. Non-invasive receptor-mediated transport strategies are also promising. Angiopep-2, which can cross the BBB via LRP1-mediated endocytosis, has been proposed as a carrier to increase the exposure of peptide drugs such as chlorotoxin conjugates in the central nervous system [81]. Pharmacokinetic studies have further confirmed that SVHRSP can cross the blood–brain barrier and be detected in cerebrospinal fluid following systemic administration [91]. However, quantitative pharmacokinetic parameters, such as the brain-to-plasma ratio, half-life in cerebrospinal fluid, and the mechanism of BBB transport (passive diffusion versus receptor-mediated transcytosis), have not been systematically characterized. Biomimetic carriers, such as exosomes, may offer advantages including low immunogenicity and good tissue penetration. In glioma models, drug-loaded exosomes achieved selective intracranial accumulation, supporting their future use in delivering scorpion peptides [98].

Critical evaluation of CNS delivery strategies for scorpion venom peptides: While the delivery strategies described above are scientifically promising, it is important to critically assess the evidence specific to scorpion venom peptides versus general drug delivery systems. Chlorotoxin-functionalized nanoparticles represent the most extensively validated scorpion peptide-based CNS delivery system. Studies have demonstrated that chlorotoxin-conjugated nanoparticles achieve receptor-mediated transcytosis across the BBB [79], brain accumulation was quantified via fluorescence imaging and ICP-MS; and functional efficacy (prolonged survival and reduced tumor volume) was observed after systemic administration in orthotopic glioma models. Toxicity assessments showed no major organ damage or hemolysis at therapeutic doses [79]. Focused ultrasound combined with microbubbles has been demonstrated to enhance BBB penetration of various therapeutic agents, but no study to date has applied this technique specifically to deliver a scorpion venom peptide into the CNS; current evidence is based on analogy from other peptides and small molecules [93]. Angiopep-2-mediated delivery has been shown to enhance BBB penetration of peptide-drug conjugates but has not been validated with a scorpion venom peptide; Angiopep-2 conjugated to unrelated payloads (e.g., resveratrol) has been used as proof-of-concept [81]. Exosome-based delivery has shown selective intracranial accumulation in glioma models, but these studies used exosomes loaded with chemotherapeutics (e.g., doxorubicin and paclitaxel), not scorpion venom peptides; demonstration with scorpion-derived cargo is lacking [98]. SVHRSP is unique among scorpion venom peptides in that its BBB penetration has been directly quantified: after systemic intraperitoneal administration in mice, SVHRSP was detected in cerebrospinal fluid and brain tissue following systemic administration, indicating that the peptide can cross the blood–brain barrier [91]. Functional efficacy (neuroprotection, reduced neuronal loss, and improved behavioral outcomes) has been demonstrated in multiple systemic administration studies [74,83,88], and no overt toxicity or immunogenicity was reported in these preclinical investigations. To recap, chlorotoxin-functionalized nanoparticles and SVHRSP itself have direct evidence supporting CNS delivery and functional efficacy, while focused ultrasound, Angiopep-2, and exosome strategies remain conceptually promising but lack validation specifically for scorpion venom peptides.

It should be noted that the quantitative values reported for focused ultrasound (2- to 10-fold BBB permeability increase, up to 50-fold peptide delivery enhancement) are derived from studies using various therapeutic agents, not specifically from scorpion venom peptides; these values represent the general potential of the technology rather than scorpion peptide-specific data.

#### Synthetic Biotechnology Approaches to Overcome Translational Obstacles

Synthetic biotechnology has emerged as a powerful toolkit to address the major translational obstacles facing scorpion venom peptide therapeutics, including low natural abundance, high manufacturing costs, metabolic instability, and immunogenicity risks. Four key biotechnological approaches are particularly relevant.

Recombinant expression systems offer a sustainable and cost-effective alternative to venom extraction. The methylotrophic yeast Pichia pastoris has been successfully used to produce active K_V_1.3 channel blockers, including Vm24, Anuroctoxin, and Ts6, at practical yields sufficient for preclinical studies [49]. This platform enables high-density fermentation, eukaryotic post-translational processing (including correct disulfide bond formation), and secretion of correctly folded peptides into the culture supernatant, simplifying downstream purification. However, expression yields for highly disulfide-bonded scorpion toxins remain variable and require optimization on a case-by-case basis. Cell-free protein synthesis (CFPS) platforms bypass the need for living cells entirely, allowing rapid production of toxic or aggregation-prone peptides that are difficult to express in conventional systems. CFPS enables high-throughput screening of engineered variants, with synthesis times reduced from weeks to hours. This approach is particularly valuable for testing large libraries of rationally designed or AI-generated sequences [99]. Chemical synthesis and engineering remains the gold standard for producing shorter scorpion venom peptides (<50 amino acids). Solid-phase peptide synthesis (SPPS) allows precise incorporation of non-natural amino acids, D-amino acids, and various modifications (cyclization, PEGylation, amidation) that enhance proteolytic stability and reduce immunogenicity [45,95]. For disulfide-rich peptides, native chemical ligation enables the assembly of larger toxins from synthetic fragments. However, the cost of SPPS scales steeply with peptide length and complexity, limiting its application for longer toxins. AI-guided design integrated with synthetic biology represents the newest frontier. Tools such as ProteinMPNN and AlphaFold3 can predict optimized peptide sequences with improved stability, selectivity, and reduced immunogenicity prior to synthesis [40,100]. Machine learning models trained on existing toxin-channel interaction data can suggest beneficial mutations that are then rapidly tested using CFPS or recombinant expression. This integrated “design–build–test” cycle accelerates the optimization of lead candidates from months to weeks.

The choice of platform depends on the specific peptide, its complexity, and the intended application. For most scorpion venom peptides, a hybrid approach combining recombinant expression for initial screening and chemical synthesis for optimized leads may be most efficient [45,99]. Across these studies, these synthetic biotechnology tools are transforming scorpion venom peptides from difficult-to-source natural products into tractable, engineerable therapeutic candidates.

### 5.2. Clinical Translation and Patent Landscape

Among scorpion venom peptide-derived drugs, synthetic chlortoxin TM-601 has the most mature clinical application. Early-phase I/II clinical trials used intracavitary injection of ^131^I-TM-601 to treat patients with recurrent high-grade gliomas, demonstrating good tolerability, limited systemic toxicity, and a relatively long tumor retention time after local administration [101]. Subsequent studies increasingly emphasize its value as a targeted ligand in imaging and payload delivery, rather than as an independent cytotoxic drug.

In analgesic drug development, Na_V_1.7-selective peptide inhibitors remain a focus of research. A review summarized engineering strategies for converting venom peptid into Na_V_1.7 antagonists and emphasized the importance of scaffold-specific optimization [102]. However, due to the high structural homology of Na_V_ channel subtypes, the risk of off-target effects on cardiac or skeletal muscle sodium channels is increased, making translational applications still very difficult. This issue is not purely theoretical: a recent study of a spider venom-derived Na_V_1.7 inhibitor found that it exhibited unexpected activity against K_V_4.2/4.3 channels, accompanied by cardiotoxicity, highlighting the need for more extensive selectivity analysis during lead compound optimization [102].

Patent activity over the past decade indicates growing interest in scorpion peptide therapies, particularly for treating neurological disorders. Among these, the thermostable peptide derivative SVHRSP, derived from the East Asian scorpion, has accumulated a relatively complete intellectual property portfolio, covering extraction methods, synthetic peptide composition, and therapeutic uses in epilepsy, Parkinson’s disease, and Alzheimer’s disease. According to official records from the Center for Drug Evaluation (CDE) of China’s National Medical Products Administration (NMPA), SVHRSP has received Clinical Trial Implicit Approval (Investigational New Drug, IND) as a Class 1.1 innovative drug. The complete regulatory information is as follows: Acceptance Number CXHL2500318; Drug Name SVHRSP Injection; Applicant/Sponsor Shenyang Wanjin Pharmaceutical Technology Co., Ltd. (Shenyang, China); Indication rapid control of epileptic seizure clusters or status epilepticus; Registration Classification Class 1.1 innovative drug; Approval Date 2025. The official regulatory record can be accessed and verified via the CDE’s public disclosure platform at https://www.cde.org.cn/main/xxgk/listpage/07edef25f1e7354bfd8490baa0ce056b (accessed on 15 March 2026).

This regulatory milestone permits the initiation of clinical trials to assess safety, tolerability, pharmacokinetics, and preliminary efficacy in humans. It is important to distinguish IND approval from marketing authorization; clinical proof-of-concept in patients has not yet been established. The ongoing clinical evaluation represents an important step toward translation but does not yet constitute proven therapeutic efficacy.

**Table 3:** Pharmacological activities, translational status, and evidence strength of representative scorpion venom peptides.

Molecule	Structural Family	Primary Target	Translational Status	Key Limitation	Citations
OD1	CSα/β (α-like)	Na_V_1.7	Preclinical lead	Cross-reactivity with Na_V_1.4/1.6	[42]
DKK2-N18W	CSα/β	Na_V_1.7	Optimized preclinical candidate	Single study; no human data	[55,56]
Charybdotoxin (ChTx)	CSα/β (K_V_-targeting)	K_V_1.3	Molecular tool	High toxicity; not therapeutic	[57]
Cvill7	CSα/α (α-KTx 2)	K_V_1.2	Discovery-stage peptide	No in vivo efficacy data; single study	[47]
BmKK2	CSα/α	K_V_1.3	Preclinical lead	Single research group; no human data	[83,99]
Chlorotoxin (TM-601)	CSβ	MMP-2; annexin A2	Clinical-stage candidate (Phase I/II)	Tumor targeting vs. direct cytotoxicity	[23,101]
SVHRSP	Synthetic derivative (heat-resistant peptide)	Multi-target (NLRP3, TLR4, NOX2, Na_V_1.6)	IND-approved (NMPA Class 1.1, Acceptance No. CXHL2500318, 2025)	Single research group; limited PK/BBB data; no human efficacy data	[83,90,91]
Pantinin-1/2	Cysteine-free linear peptide	Bacterial outer membrane LPS	Discovery-stage peptide	No in vivo efficacy data	[25]

**Note:** Translational stage definitions: Discovery-stage peptide—identified and characterized in vitro, no in vivo efficacy data. Molecular tool—validated as a research probe, not optimized for therapeutic use. Preclinical lead—in vivo efficacy demonstrated, no systematic engineering. Optimized preclinical candidate—engineered for improved stability/selectivity with PK data. IND-approved candidate—Investigational New Drug application approved, clinical trials permitted. Clinical-stage candidate—Phase I/II trials initiated. SVHRSP regulatory details: Acceptance Number CXHL2500318; Sponsor Shenyang Wanjin Pharmaceutical Technology Co., Ltd.; Indication: rapid control of epileptic seizure clusters or status epilepticus. IND approval from NMPA (China) permits Phase I clinical trials to assess safety, tolerability, and pharmacokinetics in humans. This regulatory milestone does not constitute proven therapeutic efficacy. Clinical proof-of-concept for Parkinson‘s disease, Alzheimer’s disease, or epilepsy remains to be demonstrated.

## 6. Challenges and Future Perspectives

**Figure 5:**
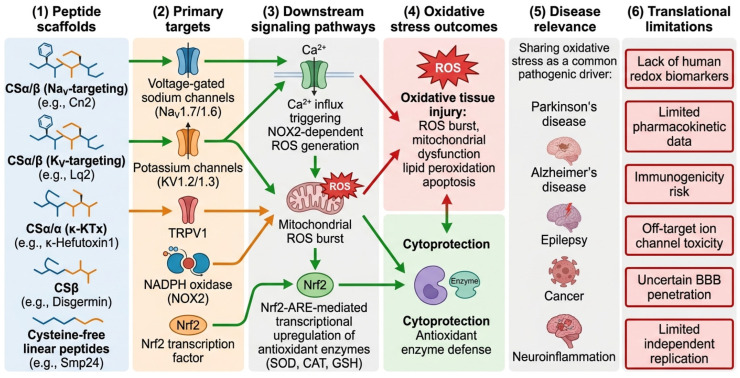
Conceptual framework linking scorpion venom peptide scaffolds to redox outcomes and translational limitations. This figure illustrates the conceptual framework connecting peptide scaffolds to primary targets, downstream signaling pathways, oxidative stress outcomes, disease relevance, and translational limitations. Arrows in different colors represent different pathways, and all arrows indicate promoting relationships. Oxidative tissue injury and cytoprotection are bidirectional, representing inhibition/induction. Red markers denote key translational barriers where evidence is currently absent or incomplete, including limited blood–brain barrier penetration data, lack of standardized human redox biomarkers, absence of independent replication for key findings (e.g., SVHRSP), and insufficient chronic toxicity or immunogenicity studies. Refer to Section 6 for detailed discussion.

Despite significant progress in scorpion venom peptide research, several critical barriers must be overcome to translate these molecules from laboratory discoveries into clinical therapeutics. Rather than providing an exhaustive list of emerging technologies, this section prioritizes the six most important translational bottlenecks: selectivity, toxicity, metabolic stability, delivery (particularly blood–brain barrier penetration), immunogenicity, and the current lack of human evidence. For each barrier, we discuss its relevance to scorpion venom peptides and potential strategies to address it (Figure 5).

### 6.1. Subtype Selectivity and Off-Target Toxicity

As discussed in Section 3.5, the high structural homology among voltage-gated ion channel subtypes poses a major selectivity challenge. Many scorpion venom peptides that are potent against the therapeutic target Na_V_1.7 also exhibit activity against cardiac (Na_V_1.5), skeletal muscle (Na_V_1.4), and central neuronal (Na_V_1.6) sodium channels, raising safety concerns [42,55]. Similarly, K_V_ channel-targeting peptides may cross-react with K_V_1.2 or K_V_1.1, potentially causing neurological or cardiac adverse effects [46,57]. Future direction: High-resolution structure-guided engineering (e.g., using cryo-EM structures or AlphaFold3 models) combined with directed evolution or rational mutagenesis will be essential to improve selectivity profiles while preserving on-target potency [40,42].

### 6.2. Metabolic Instability and Short Half-Life

Natural scorpion venom peptides are rapidly degraded by proteases in the gastrointestinal tract and bloodstream, resulting in very short in vivo half-lives (typically minutes to a few hours). This limits their practical application to injectable formulations with frequent dosing [11,92]. Future direction: Peptide engineering strategies including cyclization (e.g., BmKTX analogs), D-amino acid substitution, and Fc-fusion constructs have shown promise in extending half-life while maintaining bioactivity. These approaches should be systematically applied to lead candidates [45,76,92].

### 6.3. Blood–Brain Barrier Penetration

For neurological applications (Parkinson’s disease, Alzheimer’s disease and epilepsy), the ability to cross the BBB is a major determinant of efficacy. While chlorotoxin has been successfully used as a targeting ligand for receptor-mediated transcytosis, and SVHRSP has been detected in cerebrospinal fluid after systemic administration, quantitative data on brain penetration for most scorpion venom peptides remain limited [23,95]. Future direction: Nanoparticle encapsulation, exosome-based delivery, and receptor-targeted carriers (e.g., Angiopep-2) should be validated specifically for scorpion venom peptides, including systematic pharmacokinetic and biodistribution studies [79,95,96].

### 6.4. Immunogenicity Risk

As with all peptide therapeutics, scorpion venom peptides have the potential to elicit unwanted immune responses. The introduction of non-natural amino acids or cyclization may create neo-epitopes, potentially increasing immunogenicity rather than reducing it [99]. Future direction: Preclinical immunogenicity assessment using MHC-associated peptide proteomics (MAPP) should be integrated early into the lead optimization pipeline. In silico prediction tools (e.g., NetMHCpan) can help prioritize low-risk candidates before synthesis [99].

### 6.5. Scalable Manufacturing and Cost

Scorpion venom extraction from scorpions is low-yield, unsustainable, and raises ethical concerns. Chemical synthesis of disulfide-rich peptides is feasible but becomes expensive for longer sequences or complex folding patterns [49]. Future direction: Heterologous expression systems (e.g., Pichia pastoris) have successfully produced active K_V_1.3 blockers (Vm24, Anuroctoxin and Ts6) at practical yields. Published yields for these expressed peptides range from approximately 1 to 10 mg per liter of culture, which is sufficient for preclinical studies but would require optimization for commercial-scale production [49]. Cell-free protein synthesis platforms offer another avenue for rapid screening and production of toxic or highly engineered variants, with synthesis times reduced from weeks to hours [99].

### 6.6. Lack of Human Evidence and Validated Biomarkers

Currently, the vast majority of data supporting the antioxidant, neuroprotective, and anti-inflammatory activities of scorpion venom peptides come from cell-based and rodent models. Human data, particularly using validated redox biomarkers (e.g., plasma 8-OHdG, F2-isoprostanes and GSH/GSSG ratio), are almost entirely absent [7] TM-601 (chlorotoxin) has advanced to phase I/II trials, but primarily as a targeting/delivery agent rather than a direct therapeutic [98]. SVHRSP has received IND approval for clinical evaluation in China, but published human data are not yet available. Future direction: Future clinical trials must incorporate standardized oxidative stress biomarkers as exploratory or secondary endpoints to establish pharmacodynamic effects in humans. Independent replication of key preclinical findings (e.g., SVHRSP’s multi-target effects) by multiple research groups is also urgently needed.

### 6.7. Concluding Remarks on Future Directions

Addressing these six barriers will require a coordinated effort combining structural biology, peptide engineering, advanced formulation, and rigorous preclinical and clinical testing. Emerging technologies such as AI-guided de novo peptide design (e.g., ProteinMPNN and DiffPeptide) and improved structural prediction (AlphaFold3) offer powerful tools to accelerate this process [39,40,100]. However, these technologies must be integrated with experimental validation focused on the specific bottlenecks outlined above. With such an integrated approach, scorpion venom peptides hold promise could evolve from valuable pharmacological tools into clinically useful therapeutics for pain, cancer, and neurodegenerative disorders.

## 7. Conclusions

In summary, scorpion venom peptides represent promising experimental and preclinical scaffolds for redox-based therapeutic exploration, but it is important to recognize that they are not yet established medicines. This review establishes that their antioxidant-like activity operates through multiple distinct mechanisms: (i) Nrf2-ARE transcriptional activation, upregulating endogenous cytoprotective enzymes (SOD, CAT and GSH); (ii) direct inhibition of NOX2 assembly by blocking p47^phox^ membrane translocation, curtailing ROS generation at its source; (iii) regulation of endogenous antioxidant enzyme activity; (iv) direct radical scavenging by certain cysteine-free linear peptides; and (v), in cancer cells, the achievement of pro-oxidant effects through mitochondrial ROS generation.

However, it is crucial to note that most of the evidence for antioxidant and neuroprotective activity comes from cell-based and rodent models. Human data, particularly validated redox biomarkers (e.g., plasma 8-OHdG and GSH/GSSG ratio) in clinical settings, remain scarce. Many of the findings, particularly those related to SVHRSP, have been reported primarily by a single research group; independent replication would strengthen confidence in these observations. The synthetic peptide SVHRSP represents an interesting multi-target candidate with effects on NOX2, NLRP3, and Nrf2 pathways, and its Investigational New Drug (IND) application has been approved by China’s NMPA for clinical evaluation in epilepsy. This regulatory milestone permits Phase I clinical testing in humans but does not yet constitute proven therapeutic efficacy. Clinical proof-of-concept for Parkinson’s disease, Alzheimer’s disease, or epilepsy remains to be established. Major translational barriers remain, including subtype selectivity off-target risks, metabolic instability, immunogenicity, limited blood–brain barrier penetration, and a lack of standardized human redox biomarker data. Future research should prioritize (i) AI-guided peptide engineering to enhance selectivity and stability; (ii) nanoparticle or exosome delivery systems validated specifically for scorpion venom peptides; (iii) harmonized clinical readouts of oxidative stress; and (iv) independent replication of key findings. Looking forward, scorpion venom peptides hold promise as nature-inspired scaffolds for redox-based precision interventions. With continued innovation in structural biology, synthetic biology, and delivery technologies, these molecules have the potential to transition from pharmacological tools to therapeutic candidates, provided that rigorous selectivity, safety, and human validation studies are performed. Only a limited number of studies have been carried out from these perspectives, and further studies are urgently needed to elucidate the full spectrum of mechanisms underlying the antioxidant, neuroprotective, and immunomodulatory activities of scorpion venom peptides, as well as to assess their translational potential in human diseases.

Towards selective redox modulation: A key attribute that may distinguish certain scorpion venom peptides from conventional antioxidants is their potential to achieve cell-type-selective redox modulation. This emerging concept is based on preliminary observations from a limited number of studies. For example, the peptide S6540 induces mitochondrial ROS overproduction and caspase-independent apoptosis in A549 lung cancer cells, while SVHRSP reduces ROS levels and protects against oxidative injury in neuronal cells and microglia. The peptide Androcin 18-1 similarly shows pro-oxidant effects in U87 glioblastoma cells. As a whole, these findings suggest a hypothesis: that scorpion venom peptides might exert opposing effects—antioxidant in normal cells versus pro-oxidant in cancer cells—by leveraging differences in membrane potential, metabolic state, or surface charge distribution. However, this dual selectivity has not yet been validated across multiple peptide classes or diverse disease models. Systematic testing using broader panels of normal and cancer cells, along with mechanistic studies to identify the molecular determinants of this selectivity, is needed before this concept can be generalized. If validated, such cell-type-selective redox modulation could represent a novel strategy for precision antioxidant intervention, potentially protecting healthy tissues while sensitizing malignant cells to oxidative stress. Future efforts should focus on engineering peptides with enhanced selectivity and testing this hypothesis in patient-derived models and biomarker-stratified preclinical studies.

## Figures and Tables

**Table 2:** Major antioxidant mechanisms and evidence strength of scorpion venom peptides.

Mechanism Type	Peptide	Structural Family	Sequence/Length	Target/Pathway	Experimental Model	Evidence Strength Level	Orthogonal Validation	Direct vs. Indirect (Precise Category)	Key Limitation	Development Stage	Citation
Nrf2-ARE activation	SVHRSP	Synthetic derivative (heat-resistant peptide)	~20–30 aa (synthetic)	Nrf-2; p38 MAPK; Lnc Gm6410	PM2.5-exposed AD mouse model; *C. elegans*	Level 3	Nrf2 siRNA (in vivo)	Indirect (transcriptional)	Single research group; no human data	Preclinical	[89,90]
NOX2 inhibition	SVHRSP	Synthetic derivative (heat-resistant peptide)	~20–30 aa (synthetic)	NOX2; p47^phox^ membrane translocation	Rotenone/LPS PD mouse; BV2 microglia	Level 3	NOX2 siRNA	Direct (pathway-level: blocks NOX2 assembly)	Single research group; no PK/PD data	Preclinical	[74]
Antioxidant enzyme regulation	Smp24	Scorpion venom peptide (cysteine-containing)	24 aa (predicted)	SOD; CAT; GSH; MDA; NO	Solid Ehrlich carcinoma mouse	Level 3	None reported	Indirect (biomarker readout)	Single study; mechanism unclear	Discovery	[77]
Direct radical scavenging	TanP	Linear anionic peptide (cysteine-free)	~10–20 aa (predicted)	Free radical direct scavenging	Cell-free chemical assays	Level 1	Not applicable	Direct (cell-free assay: radical scavenging)	No in vivo data; limited structural data	Discovery	[8,74]
Pro-oxidant (cancer)	S6540	Scorpion venom peptide	N/A (from *Tityus serrulatus*)	Mitochondrial ROS; PI3K/Akt	A549 lung cancer cells	Level 2	None reported	Direct (mitochondrial ROS measurement)	No in vivo data; normal cell effects unknown	Discovery	[78]
Pro-oxidant (cancer)	Androcin 18-1	Scorpion venom peptide	18 aa (from *Androctonus crassicauda*)	Mitochondrial ROS; mitochondrial dysfunction	U87 glioblastoma cells	Level 2	None reported	Direct (mitochondrial ROS measurement)	No in vivo data; single study	Discovery	[79]

**Note:** Type of Evidence categories: Level 1 (biochemical/chemical assay, cell-free); Level 2 (cell culture only, in vitro); Level 3 (in vivo rodent model with orthogonal validation); Level 4 (in vivo efficacy with pharmacokinetic data); Level 5 (human clinical data). “Orthogonal Validation” includes genetic knockdown (siRNA/shRNA) or pharmacological inhibition. “Direct vs. Indirect” classification definitions: Direct (cell-free assay: radical scavenging): Evidence comes from cell-free chemical assays that directly measure radical-scavenging activity (e.g., DPPH, ABTS). Direct (pathway-level: blocks NOX2 assembly): Evidence suggests blockade of protein–protein interaction (p47^phox^ membrane translocation) or complex assembly, but not necessarily direct enzymatic inhibition of NOX2 catalytic activity. Direct (mitochondrial ROS measurement): Evidence directly measures mitochondrial ROS levels using fluorescent probes (e.g., MitoSOX, DCFH-DA) in live cells. Indirect (transcriptional activation): Evidence shows upregulation of Nrf2 target gene expression (e.g., SOD-3, CAT, GSH) rather than direct Nrf2 activity measurement. Indirect (biomarker readout): Evidence measures downstream antioxidant biomarkers (e.g., SOD, CAT, GSH levels, MDA, NO) as endpoints, not direct enzyme activity assays. “Key Limitation” column highlights major translational gaps. “Development Stage” categories: Discovery stage (in vitro only); Preclinical candidate (in vivo efficacy, no human data); Clinical candidate (IND approved or clinical trial). aa = amino acids; AD = Alzheimer’s disease; PD = Parkinson’s disease; LPS = lipopolysaccharide; ROS = reactive oxygen species; PK/PD = pharmacokinetics/pharmacodynamics. For S6540 and Androcin 18-1: Both peptides have been characterized in vitro only. The primary literature confirms that S6540 induces mitochondrial ROS overproduction, PI3K/Akt inactivation, and caspase-independent apoptosis in A549 cells [78]. Androcin 18-1 induces mitochondrial dysfunction and ROS accumulation in U87 glioblastoma cells [78]. In vivo efficacy, selectivity for cancer versus normal cells, and pharmacokinetic data have not yet been reported for either peptide.

## Data Availability

No new data were created or analyzed in this study. Data sharing is not applicable to this article.

## Abbreviations

The following abbreviations are used in this manuscript:

AD	Alzheimer’s disease
AMP	Antimicrobial peptide
ARE	Antioxidant response element
BBB	Blood–brain barrier
BK	Large-conductance calcium-activated potassium channel (KCa1.1)
CAR-T	Chimeric antigen receptor T cell
CAT	Catalase
Ca_V_	Voltage-gated calcium channel
CSα/α	Cysteine-stabilized α-helix/α-helix
CSα/β	Cysteine-stabilized α-helix/β-sheet
CSβ	Cysteine-stabilized β-sheet
cryo-EM	Cryo-electron microscopy
DII	Domain II
DIV	Domain IV
GSH	Glutathione
HMGB1	High-mobility group box 1
IND	Investigational New Drug
K_V_	Voltage-gated potassium channel
LPS	Lipopolysaccharide
MAPK	Mitogen-activated protein kinase
MDA	Malondialdehyde
MMP-2	Matrix metalloproteinase-2
Na_V_	Voltage-gated sodium channel
NF-κB	Nuclear factor-kappa B
NLRP3	NLR family pyrin domain containing 3
NMPA	National Medical Products Administration (China)
NMR	Nuclear magnetic resonance
NO	Nitric oxide
NOX2	NADPH oxidase 2
Nrf2	Nuclear factor erythroid 2-related factor 2
PD	Parkinson’s disease
PDB	Protein Data Bank
PI3K/Akt	Phosphoinositide 3-kinase/Protein kinase B
p47^phox^	Cytosolic subunit of NADPH oxidase 2
PTM	Post-translational modification
ROS	Reactive oxygen species
SOD	Superoxide dismutase
SOD-3	Superoxide dismutase-3
SVHRSP	Scorpion venom heat-resistant synthetic peptide
TLR4	Toll-like receptor 4
TM-601	Synthetic chlorotoxin analog
TRP	Transient receptor potential channel
TRPV1	Transient receptor potential vanilloid 1
α-KTx	Alpha-KTx (scorpion potassium channel toxin)
α-NaTx	Alpha-scorpion sodium channel toxin
β-NaTx	Beta-scorpion sodium channel toxin
κ-KTx	Kappa-KTx
